# Combining a Toggle Switch and a Repressilator within the AC-DC Circuit Generates Distinct Dynamical Behaviors

**DOI:** 10.1016/j.cels.2018.02.008

**Published:** 2018-04-25

**Authors:** Ruben Perez-Carrasco, Chris P. Barnes, Yolanda Schaerli, Mark Isalan, James Briscoe, Karen M. Page

**Affiliations:** 1 Department of Mathematics, University College London, Gower Street, WC1E 6BT London, UK; 2 Department of Cell and Developmental Biology, University College London, Gower Street, WC1E 6BT London, UK; 3 Department of Genetics, Evolution and Environment, University College London, Gower Street, WC1E 6BT London, UK; 4 Department of Fundamental Microbiology, University of Lausanne, Biophore Building, 1015 Lausanne, Switzerland; 5 Department of Life Sciences, Imperial College London, SW7 2AZ London, UK; 6 The Francis Crick Institute, 1 Midland Road, NW1 1AT London, UK

**Keywords:** gene regulatory networks, dynamical systems, multistability, oscillations, coherence, multifunctional circuits, excitable systems, synthetic biology, coherence resonance

## Abstract

Although the structure of a genetically encoded regulatory circuit is an important determinant of its function, the relationship between circuit topology and the dynamical behaviors it can exhibit is not well understood. Here, we explore the range of behaviors available to the AC-DC circuit. This circuit consists of three genes connected as a combination of a toggle switch and a repressilator. Using dynamical systems theory, we show that the AC-DC circuit exhibits both oscillations and bistability within the same region of parameter space; this generates emergent behaviors not available to either the toggle switch or the repressilator alone. The AC-DC circuit can switch on oscillations via two distinct mechanisms, one of which induces coherence into ensembles of oscillators. In addition, we show that in the presence of noise, the AC-DC circuit can behave as an excitable system capable of spatial signal propagation or coherence resonance. Together, these results demonstrate how combinations of simple motifs can exhibit multiple complex behaviors.

## Highlights

• The AC-DC circuit shows robust coexistence between oscillatory and steady expression
• The circuit allows control over the coherence of oscillations in a cell population
• The circuit shows excitable properties, allowing the spatial propagation of signals
• These suggest its prominence in development and its potential in synthetic biology

## Graphical Abstract

**Figure:**
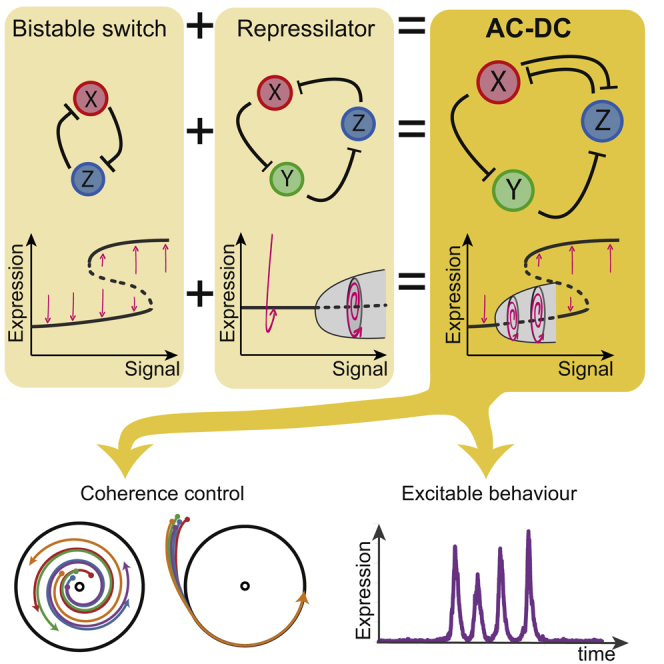

## Teaser

The AC-DC circuit, formed by the combination of a repressilator and a toggle switch, is explored in detail using dynamical systems theory and stochastic simulations. These analyses reveal that the coexistence of oscillatory and stable gene expression gives rise to novel dynamical behaviors such as control of oscillation coherence and spatial signal propagation.

## Introduction

Genetic circuits regulate biological functions in contexts that range from embryonic development to tissue homeostasis (Davidson, 2010). Accordingly, the analysis of the repertoire of functions performed by genetic circuits is central to systems biology. In some cases there is a direct relationship between the structure and operation of a circuit, such that the function—the dynamical behavior—of a circuit is evident from its topology. This has led to the classification of motifs or subnetworks based on topology and motivated the design and fabrication of artificial circuits with functions that include toggle switches, band-pass filters, memory devices, logic gates, and oscillators (Gardner et al., 2000, Elowitz and Leibler, 2000, Basu et al., 2005, Sohka et al., 2009, Ajo-Franklin et al., 2007, Siuti et al., 2013). Engineered versions of these circuits have been used to perform computation, screen for drugs, and detect and treat diseases (Daniel et al., 2013, Rubens et al., 2016, Xie et al., 2016).

Nevertheless, there is not always a one-to-one correspondence between topology and behavior. This is apparent from the analysis of even small circuits. In these cases, a small modification to such a circuit, for example the change in strength of interactions between components, leads to a qualitative change in the behavior of the circuit (Jia et al., 2017, Del Vecchio et al., 2008, Jayanthi et al., 2013, Tan et al., 2009, Prindle et al., 2014, Ingram et al., 2006). Far from being a nuisance, this has led to the concept of multifunctionality (Jiménez et al., 2017, Purcell et al., 2011)—circuits capable of qualitatively different outputs in a reduced parameter range. This poses the challenge of defining and predicting circuit behavior and emphasizes the importance of understanding the mapping between the topology of a genetic network and its dynamical behavior.

Identifying the minimal parameter changes necessary to elicit alternative behaviors from a multifunctional circuit provides insight into changes in behavior during gene network evolution and could be exploited for the engineering of circuits for synthetic biology tasks. Several studies have shed light on this problem through extensive numerical and experimental exploration of small networks targeting a specific function (Cotterell and Sharpe, 2010, Woods et al., 2016, Jiménez et al., 2017, Otero-Muras and Banga, 2016, Espinar et al., 2013, Schaerli et al., 2014). Insight from such studies is often obtained after the analysis and classification of the successful topologies in terms of the landscape of the corresponding dynamical system—sometimes called the geometrical landscape. The use of this dynamical landscape is key to revealing how different behaviors emerge, contributing to a better understanding of the mapping of topology to function (Jia et al., 2017, Strelkowa and Barahona, 2010, Süel et al., 2006, Jaeger and Monk, 2014, Verd et al., 2014).

Distilling minimal easy-to-engineer networks capable of specific functions is of paramount importance for engineering circuits for synthetic biology tasks (Schaerli et al., 2014, Purcell et al., 2010, Chau et al., 2012). Cellular resources are scarce and implementing complex behaviors within cells requires an efficient and judicious use of these (Carrera et al., 2011, Mather et al., 2013, Cookson et al., 2011). Metabolic load affects gene expression through growth-dependent effects (Klumpp et al., 2009, Scott et al., 2010, Cardinale et al., 2013), and its reduction has become a major design objective (Ceroni et al., 2015, Borkowski et al., 2016). Minimal multifunctional circuits might offer a potential route to this goal. It is therefore important to find and understand the behaviors and emergent properties that can be encoded in a reduced gene circuitry. Theoretical and computational analyses have revealed that merely combining modules with different functions does not necessarily lead to additive outcomes. Conversely, in many cases, topologies capable of multifunctional behavior cannot be explained simply as the overlap of two or more submodules (Jiménez et al., 2017). A deeper understanding of the dynamics of multifunctional circuits is needed. As there is ample evidence that real biological systems exploit multifunctionality (Verd, 2016), designing and investigating such circuits is likely to shed light on biological processes (Mathur et al., 2017).

**Figure 1:**
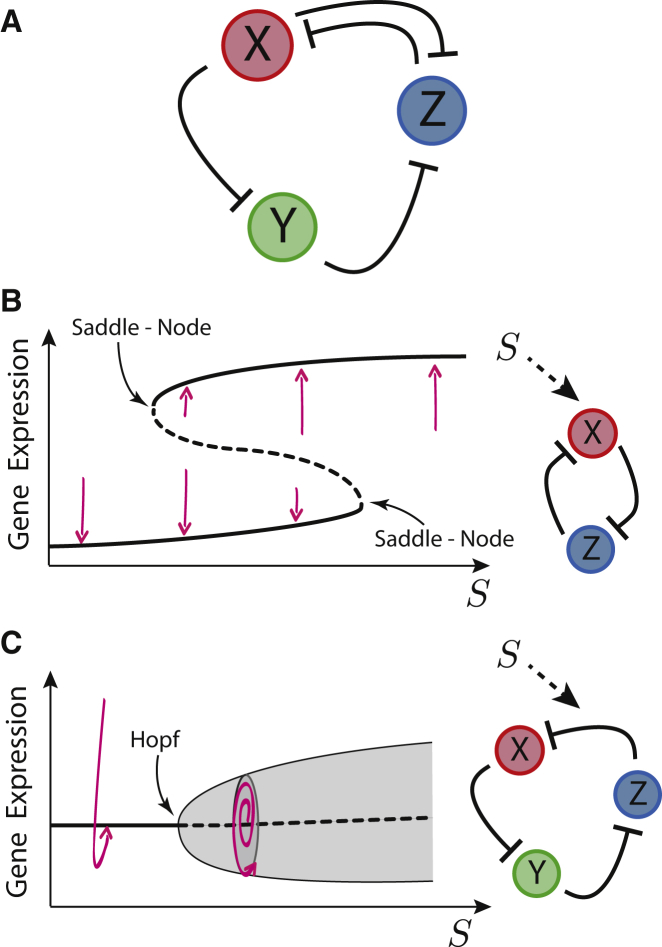
The AC-DC Circuit Is the Combination of a Bistable Switch and a Repressilator (A) AC-DC regulatory circuit. (B) Bifurcation diagram and network diagram for the bistable switch controlled by a signal *S*. The two saddle-node bifurcations position the stability range for the two stable solutions (black solid lines). There is bistability for intermediate signals, where both stable solutions are separated by an unstable steady state (dashed line). Transient trajectories (pink arrows) are sketched showing the dynamical effect of the steady states. (C) Bifurcation diagram and network diagram for the repressilator under a change of parameters controlled with an external signal *S*. The change in behavior is a Hopf bifurcation where a stable spiral (damped oscillations) (thick black solid line) becomes unstable (dashed line) giving rise to stable oscillations (shaded zone). After the bifurcation, the amplitude of the stable oscillations (delimited by thin solid lines) grows with the signal. The two possible oscillatory transients are sketched (pink arrows).

One attractive candidate for studying the coexistence and emergence of behavior in a multifunctional minimal network is the alternate current (AC)-direct current (DC) circuit (Panovska-Griffiths et al., 2013, Balaskas et al., 2012). Composed of two well-known subnetworks, the repressilator (Elowitz and Leibler, 2000, Purcell et al., 2010) and the toggle switch (Sokolowski et al., 2012, Gardner et al., 2000) (Figure 1A), the AC-DC circuit takes its name by analogy to AC and DC, since it is capable of generating oscillatory (AC) and multistable (DC) behavior (Panovska-Griffiths et al., 2013). The AC-DC circuit was originally observed in the patterning of progenitors in the vertebrate neural tube (Balaskas et al., 2012), where it is proposed to exhibit the DC behavior. Theoretical analysis revealed the potential for this circuit to generate oscillations inside a spatial pattern (Panovska-Griffiths et al., 2013) and the ability of the circuit topology to show stochastic switching between oscillations and steady-state expression (Li et al., 2012). Furthermore, Jaeger and colleagues have proposed that the gap gene system, which patterns the anterior-posterior axis of the *Drosophila melanogaster* embryo, is composed of three linked AC-DC circuits, two of which operate in the DC regime and one in the oscillatory, AC, mode (Verd, 2016).

The two subcomponents of the AC-DC circuit, the toggle switch and the repressilator, have been intensively studied, separately. Toggle switches consist of the cross-repression between the determinants of different cellular states and result in a definite choice between two outcomes. When controlled by an external signal, the toggle switch is able to produce a sharp transition between the steady states at a precise signal level (Figure 1B) (Sokolowski et al., 2012, Gardner et al., 2000). From a dynamical systems point of view, the sharp switch-like transition is the result of two saddle-node bifurcations. Each of these is characterized by the abrupt appearance of a stable and unstable steady expression state for a small change in the input signal. A consequence of this dynamical scenario is that, for a range of values of signal, both states are available and the expression state is determined by the initial gene expression. In addition, in the presence of noise, there is the possibility of switching between the stable states (Song et al., 2010, Tian and Burrage, 2006, Perez-Carrasco et al., 2016, Frigola et al., 2012).

The second component of the AC-DC circuit, the repressilator, comprises the sequential repression of three genes. In contrast to the toggle switch this provides a negative feedback loop that promotes stable oscillations. The amplitude and period of these depend on the parameters of the system (Elowitz and Leibler, 2000, Purcell et al., 2010). Changes in these parameters can lead to the disappearance of the oscillations through a Hopf bifurcation, in which the orbit in the expression space (limit cycle) shrinks, giving rise to a steady expression state. Hence coupling key parameters to an external signal can result in the repressilator becoming a switchable genetic oscillator, a property that has been extensively computationally explored (Buzzi and Llibre, 2015, Buşe et al., 2009, Purcell et al., 2010).

In this manuscript we characterize the functions of the AC-DC circuit by analyzing the phase portrait of the dynamical system. We find that oscillations and stable expression can coexist in a large region of the parameter space, and we explore the implications of this coexistence. This reveals emergent behaviors not available to the repressilator or the toggle switch individually, which allow the circuit to be used to establish coherent or incoherent oscillations. In addition, we demonstrate that, with the addition of noise, the AC-DC circuit functions as an excitable system capable of coherence resonance and spatial signal propagation.

## Results

### The Model

The expression dynamics of the AC-DC circuit can be described by taking into account the production and degradation of each gene (*X*, *Y*, and *Z*), where transcription processes are assumed to be faster than translation (Panovska-Griffiths et al., 2013). The production rate of each gene is regulated by the genetic interactions in the network and the inductive signal (*S*) that controls the behavior of the network and activates genes *X* and *Y*,

(Equation 1) $$\begin{array}{l} \dot{X}=\frac{\alpha_{X}+\beta_{X}S}{1+S+{\left(Z/z_{X}\right)}^{n_{zx}}}-X\text{,}\\ \dot{Y}=\frac{\alpha_{Y}+\beta_{Y}S}{1+S+{\left(X/x_{Y}\right)}^{n_{xy}}}-\delta_{Y}Y\text{,}\\ \dot{Z}=\frac{1}{1+{\left(X/x_{Z}\right)}^{n_{xz}}+{\left(Y/y_{Z}\right)}^{n_{yz}}}-\delta_{Z}Z\text{.} \end{array}$$

Here all the variables and parameters are non-dimensional (see STAR Methods). The non-dimensional basal production rates α_*X*_ and *α*_*Y*_ are relative to the basal production rate of gene *Z*. The signal induction is controlled by parameters *β*_*X*_ and *β*_*Y*_, while the strength and shape of the repressions are controlled by the non-dimensional factors *z*_*X*_, *x*_*Y*_, *x*_*Z*_, and *y*_*Z*_ and the exponents *n*_*zx*_, *n*_*xy*_, *n*_*xz*_, and *n*_*yz*_. Finally, the rates *δ*_*Z*_ and *δ*_*Y*_ are the relative degradation rates to the degradation of gene *X*. Similarly, the time is measured in units of time of the degradation rate of protein *X*.

The use of non-dimensional parameters allows for the study of the minimal independent set of parameters required to define the possible different dynamics of the circuit, maximizing the information obtained for any parameter fitting of the model. In the present case we are interested in finding a global behavior of the AC-DC circuit without overfitting. For this reason we performed a minimal parameter exploration looking for behaviors that exhibit a transition from non-oscillatory to oscillatory behavior through a change in the signal. In addition, we required the Hill exponents to be as low as possible to avoid numerical artifacts due to high nonlinearities. This would also ensure a set of parameters achievable in synthetic circuits.

The parameter exploration was performed using approximate Bayesian computation (Liepe et al., 2010, Liepe et al., 2014) and gave as a result the distribution of parameters necessary for observing a tunable oscillator in the AC-DC circuit. The resulting parameters (Table S1) return a consistent relationship between the parameters for different target optimizations (see STAR Methods). Namely, the basal production rate of the different genes has a marked hierarchy with *Z* being the largest, followed by genes *X* and *Y*. By contrast, both signal activation strengths are similar $\left(\beta_{X}≃\beta_{Y}\right)$. In addition, the strongest repression is that of *X* from *Z*, while the weakest is its reciprocal, from *X* to *Z*. The repressions unique to the repressilator, *x*_*y*_ and *y*_*z*_, are in between these values. All the optimizations returned a difference of at least one order of magnitude between the different repression magnitudes (*z*_*x*_ < *x*_*y*_ < *y*_*z*_ < *x*_*z*_), with clear correlations between them. Notably, a similar degradation rate was observed for all three proteins $\delta_{Y}≃\delta_{Z}≃1$. Finally, no oscillations were found when Hill exponents *n* = 2 were used, but a small increase of only one of the Hill exponents provided sufficient non-linearity to observe oscillations.

In addition to the deterministic model, it is also informative to test the behavior of the AC-DC circuit subjected to molecular intrinsic noise derived from the deterministic Equations (1) as chemical Langevin equations (Gillespie, 2000) (see STAR Methods). The inclusion of intrinsic noise has two purposes, it shows the robustness of some of the functionalities to fluctuations, while revealing new phenomena not available in a deterministic scenario.

### The AC-DC Circuit Shows Bistability between Oscillations and Steady Expression

**Figure 2:**
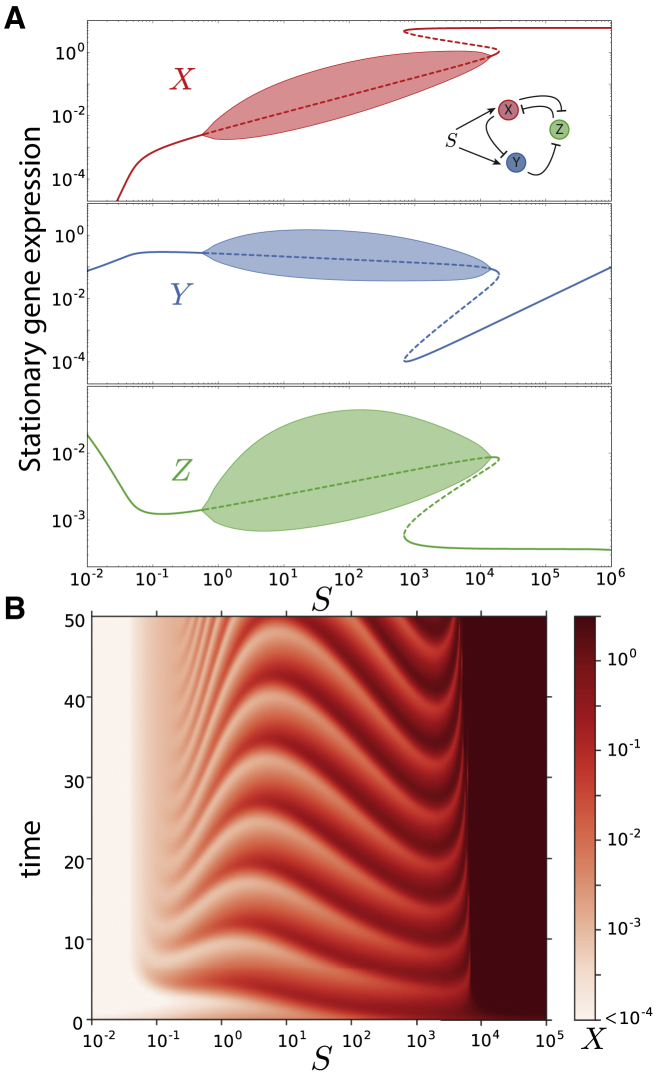
Dynamical Regimes of the AC-DC Circuit (A) Stability diagram showing the available steady states of each gene for different values of the signal *S* using the parameters of Table S1. Thick solid lines show stable steady states, dashed lines show unstable states. Shaded areas show the range of expression of oscillatory states, which are delimited by thin solid lines. The bifurcation diagram was obtained using integration and continuation techniques (Clewley, 2012). See also Figure S1. (B) Expression of gene *X* in time for different signal levels exhibiting three different dynamical regions. Initial condition is *X* = 0, *Y* = 0, *Z* = 0.

Analysis of the bifurcation diagram of the circuit reveals a mixture of the bistability from the toggle switch and the oscillatory behavior of the repressilator, in a similar way to other circuits comprising an incoherent feedback (Pfeuty and Kaneko, 2009). The Hopf bifurcation by which the oscillations arise in the repressilator transforms one of the stable states of the bistable switch into an oscillatory state that can coexist for a certain signal range with the other stable state (Figure 2; Movies S1, S2, and S3). Hence, for a given value of signal *S* both behaviors (oscillatory or stable expression) are possible. The chosen state will depend on the history of the system *S*, i.e., the system displays hysteresis. This behavior was present in 80% of the optimized parameter sets even though the parameter search optimization did not score for any kind of bistability. This suggests that it is a robust behavior arising from the network topology.

Overall, examining the bifurcation diagram shows that the behavior consisted of four different dynamical regimes for different signal ranges. For low values of the signal, there is only one possible steady state with low expression of gene *X*, resulting from a low activation of the promoters *X* and *Y* by the signal. As the signal increases, the system starts to oscillate through a Hopf bifurcation, with oscillations of small amplitude that increases with the amount of signal. For larger values of the signal a new stable state with high expression of *X* becomes available through a saddle-node bifurcation. This new state appears away from the limit cycle (steady oscillatory trajectory) without affecting it, giving rise to the bistable regime between oscillations and constant expression.

For large values of the signal the oscillations disappear. The bifurcation analysis indicated that two different mechanisms could be responsible for this. On the one hand, a Hopf bifurcation may arise collapsing the limit cycle before the second saddle-node occurs (Figure 2). On the other hand, the oscillatory state can collide with the unstable steady state produced by the saddle-node bifurcation, resulting in a homoclinic bifurcation, as previously observed by (Li et al., 2012). This gives rise to a regime in which, even though the oscillatory state is not stable, it readily generates oscillatory transients toward the steady state (Figure S1). We will consider the first case for the rest of this study; nevertheless, all the behaviors described herein are independent of the mechanism by which the oscillations disappear.

An additional regime may appear in which two constant expression steady states coexist for a wide range of signal, as expected from any bistable switch (Figure 1B). The availability of this regime depends on the location of the oscillatory region with respect to the two saddle-node bifurcations (Figure S1). Since our parameter exploration maximizes oscillatory behavior, this dynamical regime was not frequent and will not be considered in the rest of the study, which focuses instead on the coexistence between sustained oscillations and constant expression steady states.

### Coherent or Incoherent Oscillations

**Figure 3:**
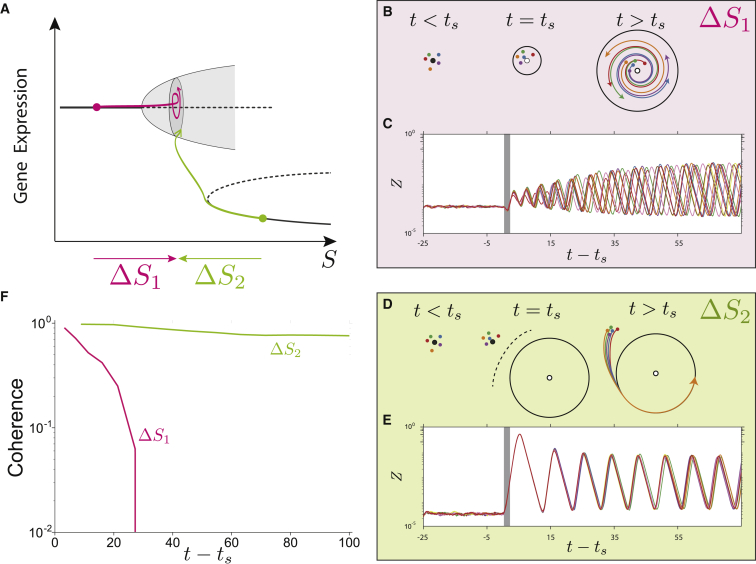
AC-DC Circuit Allows the Control of Oscillation Coherence between Different Cells (A) Schematic showing the two possible transitions toward the limit cycle. (B) Oscillations arising through the Hopf bifurcation (Δ*S*_1_) are incoherent. Diagrams show steady states and transients in the genetic expression plane. Initially, there is only one stable state (solid black circle) of constant expression, the genetic expression of different cells (colored circles) is determined by this stable state. After the signal is increased at *t* = *t_s_*, the steady state becomes unstable giving place to an unstable spiral center (solid white circle) and a stable limit cycle (black circumference). The resulting dynamical behavior for the different cells (colored arrows) follows a spiral transient toward the limit cycle. (C) Simulations of Δ*S*_1_ show the appearance of oscillations that lose their coherence by increasing the signal from *S* = 0.1 to *S* = 100 at *t* = *t_s_* for Δ*t* = 2 (gray shaded area), $\text{Ω}=10^{6}$. (D) Oscillations through the saddle-node bifurcation (Δ*S*_2_) are coherent. Diagrams show steady states and transients in the genetic expression plane. Initially, expression of cells (colored circles) are found in a stable state (solid black circle) of constant expression. After the signal is increased at *t* = *t_s_*, the steady state disappears from the plane (solid white circle) and the only attractor available is the limit cycle (black circumference), which imposes a fast expression transient toward the stable oscillations (colored lines). (E) Simulations of Δ*S*_2_ show the appearance of coherent oscillations by decreasing the signal from *S* = 105 to *S* = 100 at *t* = *t_s_* for Δ*t* = 2 (gray shaded area), $\text{Ω}=10^{6}$. (F) Comparison of the decrease in coherence in time for both signal histories Δ*S*_1_ and Δ*S*_2_ measured as $\left(\sigma_{\text{max}}-\sigma \left(t\right)\right)/\sigma_{\text{max}}$, where σ is the SD of the phase of the oscillations for 20 simulations of each mechanism and *σ*_max_ is the SD corresponding to completely incoherent oscillations.

One way of inducing oscillations through a change in signal is to increase the signal from low levels to a level above the Hopf bifurcation threshold (Δ*S*_1_). In addition, the coexistence of oscillations with saddle-node bifurcations allows for an alternative way to initiate oscillations. Starting from the stable expression state achieved at high signal levels, the oscillatory state can be reached by reducing the signal below the saddle-node bifurcation (Δ*S*_2_) (see Figure 3). Whereas in the first case a small limit cycle arises around the initial steady state, in the second case a large amplitude limit cycle is already present within the dynamical landscape when the bifurcation takes place. These differences result in different dynamical transients toward the oscillatory state.

To test these differences we performed simulations of the stochastic model starting at low or high signal and ending at the same intermediate signal value. Results show that in the first scenario—increasing signal from a low value—gives rise to asynchronous oscillations in a population of cells. Small differences in the initial phase are amplified over time. By contrast, the second scenario—decreasing signal from a high level—induces coherent oscillations in the face of noise (Figure 3; Movies S4 and S5).

This difference in behavior is a consequence of the different initial gene expression states in relation to oscillatory spiral center. In a Hopf bifurcation, oscillations arise through an attracting spiral losing its stability and becoming a repulsing spiral. Hence, oscillations originating from a Hopf bifurcation start their transient close to the unstable spiral center, and a small variation in the initial condition can lead to a substantial difference in the final oscillation phase. Small initial differences are amplified, resulting in lack of coherence of oscillations for a population of cells undergoing the bifurcation. By contrast, cells passing through the saddle-node bifurcation toward the limit cycle do so at expression levels that are far from those associated with the attracting oscillatory regime. Consequently, they have the same initial phase, and stochastic trajectories are canalized together toward the oscillatory state. In this way the AC-DC circuit, for a single set of parameters, offers the possibility to establish either coherent or incoherent oscillations in a population by choosing the appropriate signal transient. Specifically, the second mechanism is not available in the original repressilator since it requires the bistability provided by the toggle switch.

In addition to the synchrony of response, it is important to note that the saddle-node bifurcation also allows the rapid establishment of constant amplitude oscillation after the signal is reduced. Thus, the AC-DC system offers a fast mechanism to turn on and off the oscillations, which is not a feature of the repressilator. Previous studies propose robust switching exploiting quasi-stable oscillatory transients in repressilators with an even number of repressions (Strelkowa and Barahona, 2010). In contrast the AC-DC circuit exhibits the benefits of both systems, the robustness of oscillating with a stable limit cycle, and the fast switchability, in this case provided by a bifurcation occurring far from the central unstable spiral.

### The AC-DC Circuit Shows Excitability

The long-term behavior of the deterministic system in the bistable zone of the AC-DC circuit is determined by the initial conditions of the system. The set of initial conditions that are attracted to each of the two possible stable states are their respective basins of attraction. Intrinsic fluctuations in the expression levels allow the system to explore the basin of attraction, or even to cross between basins, resulting in noise-induced transitions between different cellular states (Song et al., 2010, Jia et al., 2017). In the case of the AC-DC circuit, the switching capabilities between oscillations and constant expression by intrinsic noise was analyzed by (Li et al., 2012), revealing that the frequency of switching depends on the geometry of the basin and the level of intrinsic noise. Here we explore possible effects and functionalities of this transition by altering the signal *S* and intrinsic noise levels with the system volume parameter Ω (see STAR Methods)(Gillespie, 2000, Perez-Carrasco et al., 2016).

**Figure 4:**
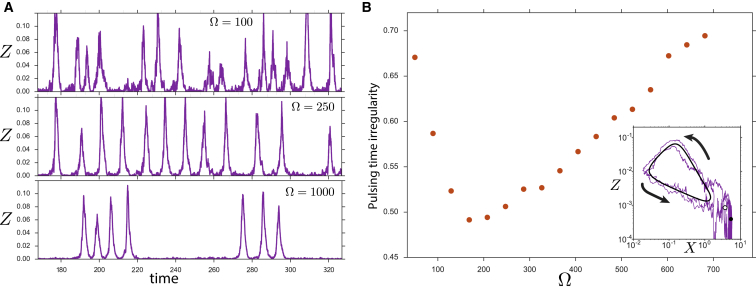
AC-DC Circuit Shows Coherence Resonance (A) Pulses of gene expression for three different noise levels. The most regular pulsing occurs for intermediate noise (Ω≃250). Simulations performed with *S* = 700. (B) Irregularity of times between pulses as a function of noise intensity. The irregularity for each value of Ω is measured as the coefficient of variation of the times between expression peaks of gene *Z* for trajectories during Δ*t* = 50,000. This illustrates minimal irregularity for intermediate Ω. Inset: Expression levels of genes *X* and *Z* during an activation that lasts for two pulses. Stochastic fluctuations drive the system far from the steady state (black circle) past the unstable steady state (white circle), driving the system around the limit cycle (black orbit). Arrows show the direction of the limit cycle. Ω = 1,000, *S* = 700.

Switching between states is not equally probable for all levels of gene expression (de la Cruz et al., 2017). In particular, once the system jumps into the oscillatory state, at least one excursion around the limit cycle is required before it can return to the constant expression state (Figure 4). Such an excursion results in the amplification of a transient fluctuation. In addition, this excursion entails a refractory period during which the system cannot be triggered again until the full cycle is finished. This pulsatile behavior reveals the excitable nature of the AC-DC circuit. Excitability has been found in other genetic systems where expression pulses have been suggested to be beneficial for the biology of the cell (Süel et al., 2006, Levine et al., 2013). In many cases the excitability arises from incoherent feedbacks (Pfeuty and Kaneko, 2009). This is the case for the AC-DC circuit where the incoherent feedback is a consequence of the superposition of its two subcircuits—the bistable switch (positive feedback) and the repressilator (negative feedback).

The frequency of the pulses depends on the noise intensity and value of the signal. In situations with low noise, the system can be trapped in the oscillatory state for more than one period (Figure 4), leading to the spike trains studied by (Li et al., 2012), which are similar to the spike trains observed in neuronal activity (Lindner et al., 2004). By contrast, if intrinsic noise is increased, the probability of observing isolated spike trains decreases. The increase in intrinsic noise results in a greater chance of exiting the limit cycle, but also a greater probability of inducing another excitation after each refractory period. This results in an increased regularity of the pulses as noise intensity augments. However, with large levels of intrinsic noise the quality of the oscillations and refractory period is disrupted, leading to a decrease in the regularity of the pulsing (Figure 4). Hence there is an optimal constructive level of noise for which the spikes become more regular. This effect, which is known as coherence resonance, is common in excitable systems and provides a way to exploit intrinsic noise for signal detection (Lindner et al., 2004, Bates et al., 2014).

**Figure 5:**
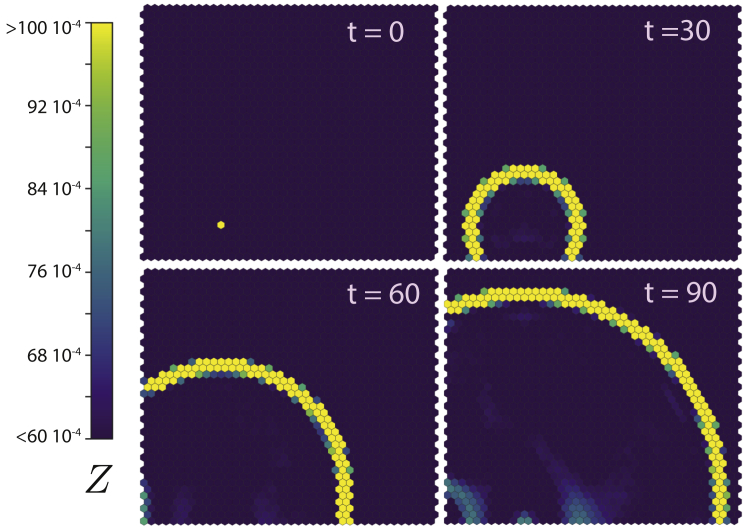
AC-DC Pulse Can Be Used as a Spatial Signal Propagation in an Array of Cells Containing the AC-DC Circuit Proteins *X*, *Y*, and *Z* can diffuse intercellularly. Cells are under signal *S* = 1,000 (bistable regime) and initially set at the constant expression steady state except one cell that is perturbed away from the steady state. This perturbation initiates a wave that propagates through the field. Ω = 10^5^, *D* = 0.1 (see more details in the Supplemental Information).

The excitable nature of the AC-DC circuit might also be relevant to other functions, such as signal propagation in a tissue. A cell signaling to neighboring cells can be excited to undergo a pulse that will, in turn, excite neighboring cells and so on. The intensity and transient and refractory periods of the pulse not only contribute to the excitation of neighboring cells but also inhibit the reactivation of the recently excited cells, resulting in a spatially propagating pulse over the tissue. To test this possibility, we performed a series of numerical assays in a simulated tissue where one or more of the proteins forming the AC-DC circuit diffuse between cells. To initiate the system, bistable cells are set in the constant stable expression state. In this scenario, the induced excitation of one cell leads to a propagating front in which cells are excited sequentially, returning afterward to the initial constant expression state (Figure 5; Movie S6). During this period of time, the transient expression along the limit cycle is high enough to deliver the pulse to the neighboring cells. The width and velocity of the propagating front can be controlled with the noise intensity or the diffusion coefficient, as well as the signaling mechanism between cells. In addition, for a high enough level of noise, spontaneous propagation waves can also occur, as well as dynamical patterning of the system (see Movies S6, S7, S8, and S9). Similar results would be expected with more elaborate signaling pathways in which transmembrane receptors are involved in the transmission of the signal (Mathur et al., 2017, Jiménez et al., 2017, Formosa-Jordan et al., 2012).

## Discussion

We have explored the behavior of the multifunctional AC-DC circuit, showing that the coexistence between bistability and oscillations elicit novel dynamics that are unavailable to either of its constituent parts. These provide a mechanism to rapidly switch between a rhythmic and steady expression regime and suggests a way in which coherent oscillations can be induced into an ensemble of otherwise noisy oscillators. In addition, we demonstrate the excitable dynamics of the system that result in the potential for coherence resonance and spatial signal propagation. Notably, these behaviors are accessible for the same range of parameters, supporting the idea that the circuit represents a versatile genetically encoded network well suited for a range of functions.

Although not apparent from the structure of the network, the various functions of the AC-DC circuit become evident from an inspection of the geometry of its dynamical landscape. As has been shown in previous studies, the position and nature of the attractors of the system provide substantially greater insight than a simple analysis of the topology of the network (Jiménez et al., 2017, Cotterell and Sharpe, 2010, Süel et al., 2006, Strelkowa and Barahona, 2010, Jia et al., 2017, Jaeger and Monk, 2014, Verd et al., 2014, Pfeuty and Kaneko, 2009). For the AC-DC circuit, the shape of the dynamical landscape is created by the combination of saddle-node bifurcation arising from the toggle switch and the Hopf bifurcation of the repressilator. Moreover, transitions such as the sudden destabilization of oscillations through the homoclinic bifurcation, can be anticipated from examining the structure of the dynamical landscape. This highlights the application and importance of dynamical systems tools for identifying and explaining the behavior of even relatively simple circuits. It also raises the possibility that similar dynamical behaviors might be present in other circuits composed of incoherent feedbacks that share similar attractor landscapes (Pfeuty and Kaneko, 2009, Krishna et al., 2009).

Besides its potential applications in synthetic biology (see Box 1), the AC-DC circuit also offers insight into genetic circuits involved in tissue development. During vertebrate embryogenesis, coordinated gene expression oscillations—the segmentation clock—in the posterior cells of the body generate a rhythmic spatial pattern that subdivides the embryonic trunk into morphological segments (Hubaud and Pourquié, 2014). This involves a still poorly defined genetic oscillator within posterior cells. Notably, individual cells appear to behave as autonomous oscillators that, when isolated from the embryo, produce transient stochastic periods of oscillations (Webb et al., 2016). In addition, a recent experimental study has revealed the excitable nature of such oscillations (Hubaud et al., 2017). This behavior features the same properties found in the excitable regime of the AC-DC circuit in which a limit cycle and steady expression state coexist, suggesting that the AC-DC circuit could provide a model for the process. In a different tissue, the *Drosophila* blastoderm, the dynamics of three linked AC-DC gene circuits have been proposed to characterize the regulatory network that patterns the anterior-posterior axis (Verd, 2016). In this case, the dynamical transients of the AC-DC circuit are suggested to tune the position of the boundaries in time. Moreover, the presence of AC-DC dynamics in this gene network has been suggested later on to reconcile differences between short and long germband insects (Clark, 2017). In short germband insects, rhythmic expression of genes is associated with the gradual extension of the body axis. By contrast in long germband insects, such as *Drosophila*, the trunk is patterned simultaneously without cyclic expression of the patterning genes. The bistability between a stable steady state and a limit cycle and the possibilities to transition smoothly between regimes with a change of one parameter suggests a route for the evolutionary transition of the underlying gene-regulatory network (Verd, 2016). In this view, the multifunctionality of the AC-DC circuit contributes to the evolvability of the circuit and exemplifies how the competing demands of biological mechanisms to be both robust and adaptable can be satisfied. Accordingly, studies of genetically encoded circuits such as the AC-DC network provide insight into the design principles of regulatory mechanisms that characterize biology.

Box 1 **Applications of the AC-DC Circuit in Synthetic Biology**

Of particular interest for the AC-DC circuit is the multistability and the ability to control switching between the different behaviors by changing a single external signal. The availability of wide regions of parameter space, in which these behaviors take place, makes the AC-DC circuit an attractive target for potential applications in synthetic biology. In particular, the problem of generating synchronized ensembles of oscillators is a challenge. Considerable efforts have been made to solve this problem either by engineering away noise or relying on quorum sensing (McMillen et al., 2002, Garcia-Ojalvo et al., 2004, Kobayashi et al., 2004, Kuznetsov et al., 2006, Danino et al., 2010, Nikolaev and Sontag, 2016, Potvin-Trottier et al., 2016) The AC-DC circuit offers a novel strategy that relies on the bistable oscillatory regime, producing a favorable robust dynamical transient toward the oscillations. Moreover, the circuit offers the possibility of exhibiting different degrees of coherence in response to a single triggering signal. This offers a new tool for synthetic biology to control the heterogeneity of gene expression in a population of cells.

Pulsatile excitations are also a property exploited in several biological situations. The behavior is reminiscent of bet-hedging strategies that have been proposed to optimize responses to external inputs or maximize the use of limited resources by controlling the time at which nutrient demanding physiological processes occur (Levine et al., 2013, Süel et al., 2006, Liu et al., 2017). From this perspective, the AC-DC circuit provides a mechanism to explore different excitable regimes by changing the external signal without the need to control the levels of intrinsic noise by altering the copy number or degradation rates of the system (Hilborn et al., 2012, Niederholtmeyer et al., 2015).

Moreover, the AC-DC circuit exhibits pulsatile properties. In other circuits the excitability arises from unstable excitable transients or through a subcritical Hopf bifurcation (Süel et al., 2006,; Hilborn et al., 2012). In the AC-DC circuit, the separation of the saddle-node bifurcation that initiates oscillations from the amplitude of the limit cycle allows for parameterizations in which both properties of the bistable region can be tuned independently to control the different features of the pulses. Similar distinctions are found in excitable systems, such as those associated with neuronal action potentials (Izhikevich, 2000, Lindner et al., 2004), raising the possibility of combining current advances in neuronal networks and excitable media with synthetic genetic circuits. In particular, the tunability of the excitable properties allows for signal propagation across a population of cells with different velocities and intensities that can be slower than the typical production and degradation rates of the molecular components of the circuit. In addition, it suggests the possibility of exploiting coherence resonance for signal detection. This property results when an increase in noise intensity produces an increased probability of crossing a critical threshold in excitable systems. In both neurological and manufactured systems this can be used to increase the signal-to-noise ratio and therefore to enhance the detection of weak signals (Lindner et al., 2004, Bates et al., 2014). The capacity of the AC-DC circuit for coherence resonance raises the possibility of exploiting this feature in the design of biosensing applications in synthetic biology (Van Der Meer and Belkin, 2010).

## STAR★Methods

### Key Resources Table

**Table:** 

REAGENT or RESOURCE	SOURCE	IDENTIFIER
**Software and Algorithms**		

ABC – SysBio	Liepe et al., 2010	http://www.theosysbio.bio.ic.ac.uk/resources/abc-sysbio/
PyDSTool	Clewley, 2012	http://www2.gsu.edu/∼matrhc/PyDSTool.htm RRID:SCR_014771

### Contact for Reagent and Resource Sharing

Further information and requests for resources and reagents should be directed to and will be fulfilled by the Lead Contact, Ruben Perez-Carrasco.

### Method Details

#### Non-dimensional Equations

The nondimensional equations [1] result from the dimensional Hill function regulation,

(Equation 2) $$\begin{array}{l} \frac{\text{d}\tilde{X}}{\text{d}\tilde{t}}=\frac{{\tilde{\alpha }}_{X}+{\tilde{\beta }}_{X}S}{1+S+{\left(\tilde{Z}/{\tilde{z}}_{X}\right)}^{n_{zx}}}-{\tilde{\delta }}_{X}\tilde{X}\\ \frac{\text{d}\tilde{Y}}{\text{d}\tilde{t}}=\frac{{\tilde{\alpha }}_{Y}+{\tilde{\beta }}_{Y}S}{1+S+{\left(\tilde{X}/{\tilde{x}}_{Y}\right)}^{n_{xy}}}-{\tilde{\delta }}_{Y}\tilde{Y}\\ \frac{\text{d}\tilde{Z}}{\text{d}\tilde{t}}=\frac{{\tilde{\alpha }}_{Z}}{1+{\left(\tilde{X}/{\tilde{x}}_{Z}\right)}^{n_{xz}}+{\left(\tilde{Y}/{\tilde{y}}_{Z}\right)}^{n_{yz}}}-{\tilde{\delta }}_{Z}Z\text{.} \end{array}$$

Measuring time in units of the degradation rate of protein *X*, all the temporal variables can be nondimensionalized as,

(Equation 3) $$\delta_{Y}=\frac{{\tilde{\delta }}_{Y}}{{\tilde{\delta }}_{X}}\text{,}\;\delta_{Z}=\frac{{\tilde{\delta }}_{Z}}{{\tilde{\delta }}_{X}}\text{,}\;t=\tilde{t}\delta_{X}\text{.}$$

Similarly, concentrations and rates can be non-dimensionalized using the timescale of ${\tilde{\delta }}_{X}$ and the production rate ${\tilde{\alpha }}_{Z}$.

(Equation 4) $$\alpha_{X}=\frac{{\tilde{\alpha }}_{X}}{{\tilde{\alpha }}_{Z}}\text{,}\;\alpha_{Y}=\frac{{\tilde{\alpha }}_{Y}}{{\tilde{\alpha }}_{Z}}\text{,}\;\beta_{X}=\frac{{\tilde{\beta }}_{X}}{{\tilde{\alpha }}_{Z}}\text{,}\;\beta_{Y}=\frac{{\tilde{\beta }}_{Y}}{{\tilde{\alpha }}_{Z}}$$

(Equation 5) $$z_{x}=\frac{{\tilde{z}}_{x}{\tilde{\delta }}_{X}}{{\tilde{\alpha }}_{Z}}\text{,}\;x_{y}=\frac{{\tilde{x}}_{y}{\tilde{\delta }}_{X}}{{\tilde{\alpha }}_{Z}}\text{,}\;x_{z}=\frac{{\tilde{x}}_{z}{\tilde{\delta }}_{X}}{{\tilde{\alpha }}_{Z}}\text{,}\;y_{z}=\frac{{\tilde{y}}_{z}{\tilde{\delta }}_{X}}{{\tilde{\alpha }}_{Z}}$$

(Equation 6) $$X=\frac{\tilde{X}{\tilde{\delta }}_{X}}{{\tilde{\alpha }}_{Z}}\text{,}\;Y=\frac{\tilde{Y}{\tilde{\delta }}_{X}}{{\tilde{\alpha }}_{Z}}\text{,}\;Z=\frac{\tilde{Z}{\tilde{\delta }}_{X}}{{\tilde{\alpha }}_{Z}}$$

The signal *S* is also measured in arbitrary units. Since *S* is a control parameter to control the dynamics properties of the system, the results will hold for any non-linear relationship between concentration of inducer and *S*.

For the stochastic Chemical Langevin Equation, the parameter Ω relates the non-dimensional expression levels with actual number of proteins (*N*_*X*_, *N*_*Y*_, *N*_*Z*_) as,

(Equation 7) $$N_{X}=\frac{X{\tilde{\alpha }}_{Z}\text{Ω}}{{\tilde{\delta }}_{X}}\text{,}\;N_{Y}=\frac{Y{\tilde{\alpha }}_{Z}\text{Ω}}{{\tilde{\delta }}_{X}}\text{,}\;N_{Z}=\frac{Z{\tilde{\alpha }}_{Z}\text{Ω}}{{\tilde{\delta }}_{X}}\text{.}$$

#### Parameter Fitting

The parameter exploration was carried out using Bayesian sampling techniques through the Approximate Bayesian Computation (ABC) using ABC-SysBio software (Liepe et al., 2010). The score functions, d(), are minimal for the optimal behavior scored. They were designed to capture a change from stable steady state to oscillations. This was evaluated on trajectories for each parameter set under the induction of two different signal values *S*_*DC*_ and *S*_*AC*_.

First the network is induced by a low signal $\left(S=S_{DC}\right)$ for Δ*t* = 50. Allowing a transient of Δ*t* = 30 (see Figure S2), after which a constant response in time is scored:

(Equation 8) $$d_{DC}\left(X\left(t\right)\right)=M_{DC}+2\frac{\text{max}\left(X\left(t\right)\right)-\text{min}\left(X\left(t\right)\right)}{\text{max}\left(X\left(t\right)\right)+\text{min}\left(X\left(t\right)\right)}$$

Where *M*_*DC*_ is the number of minima found, penalising oscillations. The second term of *d*_*DC*_ penalises transients far from a steady expression.

The constant regime is perturbed by increasing the signal to a new value by multiplying it by a factor *σ*, *S*_*AC*_ = *S*_*DC*_*σ* (*σ* > 1) applied for Δ*t* = 100. The factor *σ* was also allowed to vary during the parameter exploration. During this second period, the goodness of the oscillations was evaluated favouring large oscillation amplitudes, and penalising a non-constant amplitude in time:

(Equation 9) $$d_{AC}\left(X\left(t\right)\right)=\left\{ \begin{array}{cc} \frac{1}{M_{AC}}+2 & M_{AC}<4\\ \left|\frac{A_{M}-A_{M-1}}{A_{M-1}}\right|+2\frac{\text{min}\left(X\left(t\right)\right)}{\text{max}\left(X\left(t\right)\right)\text{+min}\left(X\left(t\right)\right)} & M_{AC}>4\text{,} \end{array}\right.$$

where *M*_*AC*_ is the number of maxima found after a transient of Δ*t* = 20 region, and *A*_*M*_ and *A*_*M−1*_ are the amplitudes of the last and the previous to last full oscillations (see Figure S2). Both parameters *S*_*AC*_ and *σ* were treated as free parameters of the optimisation.

Finally, in order to reduce artefacts arising from the choice of high Hill exponents, all the Hill exponents were set to *n* = 2, varying only one exponent that is penalised to have higher values in circuits that already have a low score,

(Equation 10) $$d_{Hill}\left(n_{i}\right)=\{\begin{array}{cc} 2 & d_{AC}+d_{DC}>2\\ \left|\frac{2-n_{i}}{3}\right| & d_{AC}+d_{DC}\leq 2 \end{array}$$

The distance used to infer the parameters used in the the current study (Table S1, and Figure S3) was,

(Equation 11) $$d=d_{DC}\left(X\right)+d_{AC}\left(X\right)+d_{Hill}\left(n_{zx}\right)\text{,}$$

where the minimisation was run for 20 generations of the ABC optimisation and the expected behavior started to arise beyond generation 10 of the ABC optimisation. To test possible overfitting resulting from the functions used, alternative functions were designed resulting in similar results analysed during different generations of the algorithm, some examples are shown in Table S2 where,

(Equation 12) $$d_{1}=d_{DC}^{2}\left(Y\right)+d_{AC}^{2}\left(Y\right)+d_{Hill}^{2}\left(n_{zx}\right)\text{,}$$

(Equation 13) $$d_{2}=d_{DC}\left(X\right)+d_{AC}\left(Y\right)+d_{Hill}\left(n_{yz}\right)\text{.}$$

#### Stochastic Expression

The stochastic dynamics of expression was studied using the Chemical Langevin equation resulting from taking into account the stochastic nature of the production and degradation events (Gillespie, 2000) as:

(Equation 14) $$\dot{X}=f_{X}\left(Z\text{,}\;S\right)-X+\sqrt{f_{X}^{2}\left(Z\text{,}\;S\right)+X^{2}}\xi_{X}\left(t\right)\text{,}$$

$$\dot{Y}=f_{Y}\left(X\text{,}\;S\right)-\delta_{Y}Y+\sqrt{f_{Y}^{2}\left(X\text{,}\;S\right)+\delta_{Y}^{2}Y^{2}}\xi_{Y}\left(t\right)\text{,}$$

$$\dot{Z}=f_{Z}\left(X,Y\right)-\delta_{Z}Z+\sqrt{f_{Z}^{2}\left(X\text{,}\;Y\right)+\delta_{Z}^{2}Z^{2}}\xi_{Z}\left(t\right)\text{,}$$

where *f*_*X*_, *f*_*Y*_, and *f*_*Z*_ are the production terms of equations.(1) and $\xi_{i}$ are uncorrelated white Gaussian noises of zero mean and autocorrelation $\langle \xi_{i}\left(t\right)\xi_{i}\left(t'\right)\rangle ={\text{Ω}}^{-1}\delta \left(t-t'\right)$, where δ(t−t') is Dirac’s delta and Ω is the system volume, relating expression concentrations with number of proteins.

#### Cell Lattice Diffusion

Spatially extended simulations for the genetic expression propagation were carried out implementing an array of hexagonal cell of unit length. One or more of the proteins forming the AC-DC circuit are allowed to diffuse between neighbours using a discrete Laplacian that for gene *X* of the *i*-th cell reads

(Equation 15) $${\dot{X}}_{i}=D\left({\langle X\rangle }_{\left\{i\right\}}-X_{i}\right)\text{,}$$

where *D* is the intercellular diffusion coefficient and ${\langle \cdot \rangle }_{\left\{i\right\}}$ stands for the average expression of the target gene among of all the neighbouring cells of cell *i*.

## Supplemental Information

Document S1. Figures S1–S3 and Tables S1 and S2

Movie S1. Expression of Gene *X* in a Cellular Lattice in Time under a Gradient of Signal $S=10^{-7x/80-2}$, Related to Figure 2 The gradient is equivalent to the signal set in Figure 2, using the same color code. Initial condition is *X* = 0, *Y* = 0, *Z* = 0.

Movie S2. Resulting Spatiotemporal Profile of Movie S1 Separating the Individual Expression of Each of the Three Genes *X*, *Y*, and *Z*, Related to Figure 2

Movie S3. Gene Expression for a Spatiotemporal Signaling Gradient in a Cellular Lattice, Related to Figure 2 The signal starts at *S* = 0 along the whole tissue and increases temporally building the same gradient as in Movie SI Movies. $S=10^{-7x/80-2}{\text{e}}^{-2/t}$.

Movie S4. Time Evolution of Six Different Stochastic Trajectories for the Signaling ΔS1 for the Same Parameters and Time Protocol of Figure 3, Related to Figure 3 The signal is fixed at *S* = 0.1 and is maintained until *t* = 25, when it is increased gradually during Δ*t* = 2 until reaching a final signal *S* = 100 that is maintained until *t* = 100. During that time the system undergoes a Hopf bifurcation setting the expression state close to the spiral center. This entails a divergence between the different trajectories that results in a lack of coherence in the oscillations.

Movie S5. Time Evolution of Six Different Stochastic Trajectories for the Signaling ΔS2 for the Same Parameters and Time Protocol as Figure 3, Related to Figure 3 The signal is fixed at *S* = 10^5^ and is maintained until *t* = 25, when it is increased gradually during Δ*t* = 2 until reaching a final signal *S* = 100 that is maintained until *t* = 100. The system undergoes a saddle-node bifurcation setting the expression state of the different trajectories at a similar position far from the limit cycle. The cycle attracts all the trajectories following the same slow manifold resulting in coherent convergence to the oscillatory behavior.

Movie S6. Expression of Gene *Z* in an Array of Cells Where the AC-DC Proteins Can Diffuse with a Signal *S* = 1,000 (Bistable Regime), Related to Figure 5 Cells are initially at the constant expression steady state except one cell that is perturbed away from the steady state. That cell undergoes a cycle around the limit cycle increasing the levels of *Z* that diffuses to the neighboring cells exciting them. The result is a propagating front of activation of gene *Z*. Ω = 10^5^, *D* = 0.1 (see more details in the Supplemental Information).

Movie S7. Expression of Gene *Z* in an Array of Cells Where the AC-DC Proteins Can Diffuse with a Signal *S* = 1,000 (Bistable Regime), Related to Figure 5 Cells are initially at the constant expression steady state except one cell that is perturbed away from the steady state. A larger diffusion coefficient results in a wider and faster front than in Movie S6. Ω = 10^7^, *D* = 0.2 (see more details in the Supplemental Information).

Movie S8. Expression of Gene *Z* in an Array of Cells Where the AC-DC Proteins Can Diffuse with a Signal *S* = 1,000 (Bistable Regime), Related to Figure 5 Cells are initially at the constant expression steady state except one cell that is perturbed away from the steady state. A larger intrinsic noise than in Movies S6 and S7 results in a front that leaves behind an excited media where intrinsic noise results in a dynamical spatial pattern. Ω = 2000, *D* = 0.2 (see more details in the Supplemental Information).

Movie S9. Expression of Gene *Z* in an Array of Cells Where the Protein *Z* Can Diffuse Intercellularly with a Signal *S* = 1,000 (Bistable Regime), Related to Figure 5 Cells are initially at the constant expression steady state except one cell that is perturbed away from the steady state. Allowing only a single protein to diffuse still exhibits the excitable wave reducing its width and speed even considering a higher intrinsic noise and diffusion coefficient. Ω = 100, *D* = 0.5 (see more details in the Supplemental Information).

Document S2. Article plus Supplemental Information
