# Supplementary material for: Combining a Toggle Switch and a Repressilator within the AC-DC Circuit Generates Distinct Dynamical Behaviors
Source: Cell Syst. 2018 Apr 25;6(4):521–530.e3. doi: 10.1016/j.cels.2018.02.008 (PMC5929911; doi:10.1016/j.cels.2018.02.008)
Supplement: Document S1. Figures S1–S3 and Tables S1 and S2 [file mmc1.pdf]

Cell Systems, Volume *6*

Supplemental Information

Combining a Toggle Switch and a Repressilator within the AC-DC Circuit Generates Distinct Dynamical Behaviors

Ruben Perez-Carrasco, Chris P. Barnes, Yolanda Schaerli, Mark Isalan, James Briscoe, and Karen M. Page

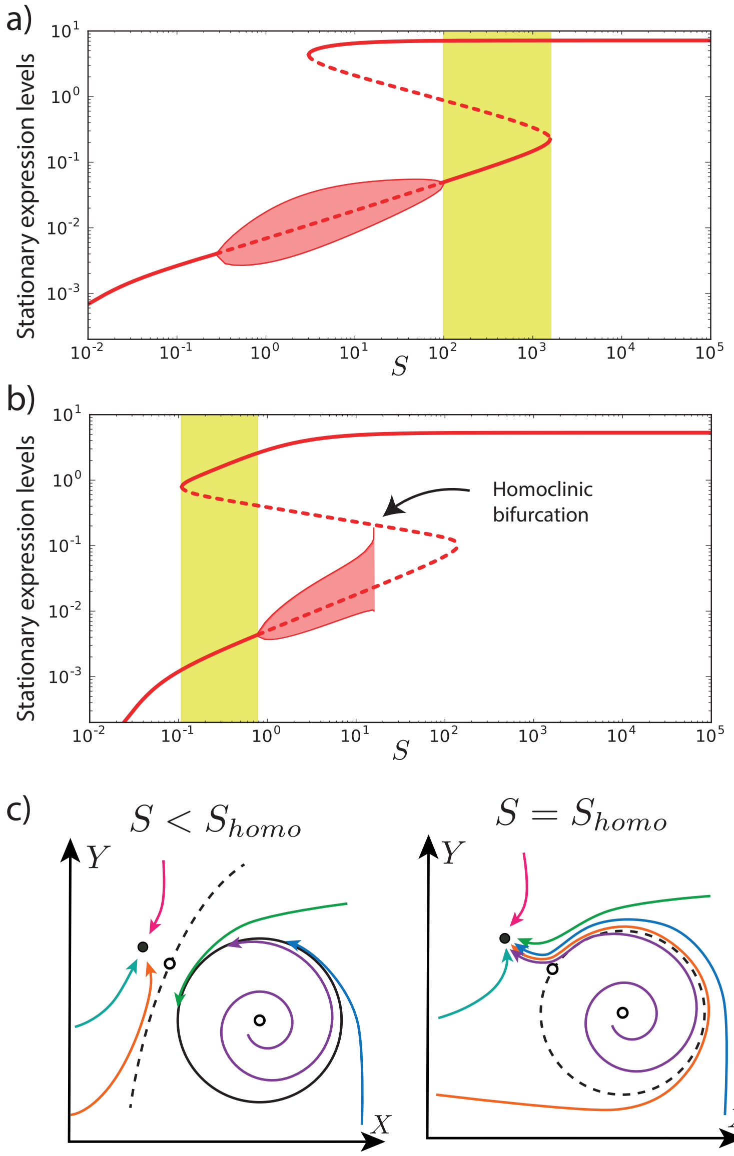

Figure S.1: Related to Figure 2. a) and b): Bifurcation diagrams showing bistability between two stable points for a wide range of signal (*yellow shaded zones*). This bistability is determined by the relative position of the saddle node bifurcations with respect to the Hopf bifurcation. This allows a bistable region between points, after (a) or before (b) the Hopf bifurcations. b) At high signals, stable oscillations can disappear through a homoclinic bifurcation instead of a Hopf bifurcation where the limit cycle collides with the unstable steady state at a signal $S_{homo} \simeq 16$. c) Scheme showing the attractor change at the homoclinic bifurcation. When the unstable saddle point (white point in the dashed line), collides with the limit cycle (solid circle), the cycle becomes unstable (dashed circle) and sustained oscillations are not available anymore. The only stable steady state remaining is the constant expression node that now attracts eventually all the expression trajectories except those starting precisely on the limit cycle. Parameters used for a) are $\alpha_x = 1.5$,$\alpha_y = 0.31$, $\beta_x = 7.17$, $\beta_y = 14.9$, $n_{xy} = 2.7$, $\delta_y = 1.31$, $\delta_z = 1.41$, $z_x = 1.75 \cdot 10^{-3}$, $x_y = 2.0 \cdot 10^{-3}$,$x_z = 0.19$, $y_z = 0.13$. Parameters used for b) are $\alpha_x = 0.486$,$\alpha_y = 0.252$, $\beta_x = 5.31$, $\beta_y = 6.2$, $n_{zx} = 2.8$, $\delta_y = 1.07$, $\delta_z = 2.0$, $z_x = 1.94 \cdot 10^{-3}$, $x_y = 1.4 \cdot 10^{-3}$,$x_z = 0.038$, $y_z = 0.097$.

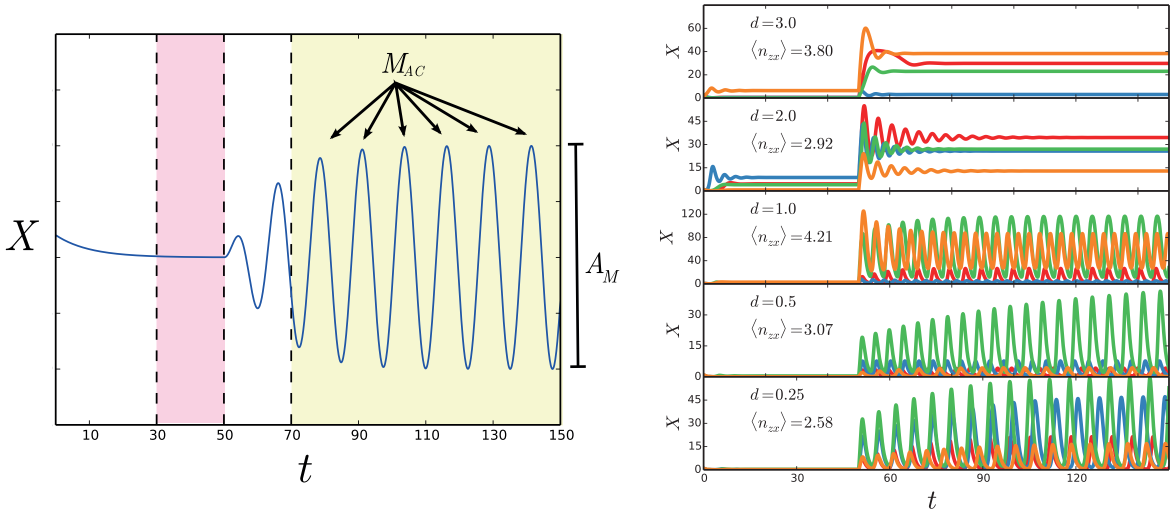

Figure S.2: Related to STAR Methods. Left) Target behavior used in the score function, evaluated at the shaded areas. The number of expression maxima at high signal M_{AC} and amplitude of the last oscillations A_M is used to compute the quality of oscillations. Right) Example results of behavior of the circuit under the two-signal protocol for different score results during the optimisation process.

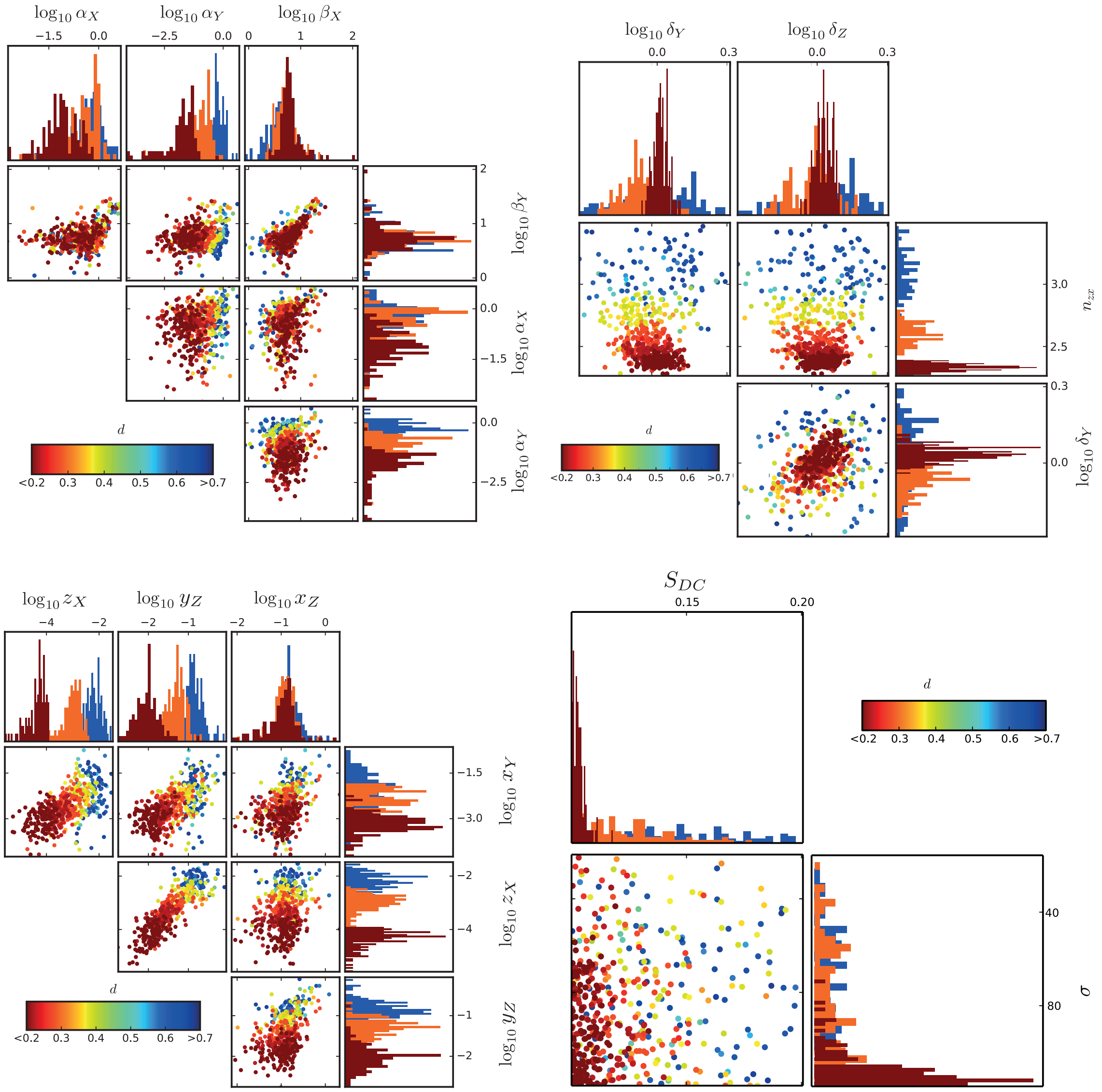

Figure S.3: Related to STAR Methods. Resulting parameter inference. Histograms correspond with the results of generations 10 (*blue*), 15 (*orange*) and 20 (*dark red*), colored by the average distance score of the generation. Scatter plots contain the sample used in generations 5,8,11,14, and 17. Parameters are grouped in 4 categories to make easier the comparison between different magnitudes.

Parameter	$\bar{x}\pm$ s.d	mode
α_X	$(15 \pm 14)10^{-2}$	$3.9 \cdot 10^{-2}$
α_Y	$(2.7 \pm 2.1)10^{-2}$	$4.3 \cdot 10^{-3}$
β_X	5.9 ± 0.8	6.1
β_Y	5.4 ± 1.2	5.7
δ_Y	1.07 ± 0.08	1.05
δ_Z	1.12 ± 0.09	1.04
z_X	$(6.4 \pm 4.3)10^{-5}$	$1.3 \cdot 10^{-5}$
y_Z	$(11 \pm 4)10^{-3}$	$11 \cdot 10^{-3}$
x_Z	$(12 \pm 5)10^{-2}$	$12 \cdot 10^{-2}$
x_Y	$(8.3 \pm 4.2)10^{-4}$	$7.9 \cdot 10^{-4}$
n_{zx}	2.34 ± 0.04	2.32

Table S.1: Related to Figure 2. Optimal parameters resulting from the last 300 points of the ABC fitting (generations 18-20). Each row shows the mean, standard deviation, and position of the peak of the distribution (mode). Rest of the Hill exponents were fixed to 2. For details see SI.

	d	$d^{(13)}$	d^*	d_1	d_2
α_X	0.57 ± 0.37	0.43 ± 0.31	0.64 ± 0.37	0.60 ± 0.39	0.60 ± 0.39
α_Y	0.21 ± 0.19	0.066 ± 0.055	0.18 ± 0.14	0.19 ± 0.14	0.23 ± 0.17
β_X	4.9 ± 1.3	5.4 ± 1.1	5.1 ± 1.5	4.9 ± 1.4	5.1 ± 1.3
β_Y	5.7 ± 1.0	5.2 ± 1.3	5.7 ± 1.2	5.6 ± 1.1	5.5 ± 1.1
δ_Y	0.93 ± 0.18	1.0 ± 0.1	0.94 ± 0.17	1.02 ± 0.27	1.00 ± 0.27
δ_Z	1.04 ± 0.22	1.1 ± 0.1	1.06 ± 0.21	1.05 ± 0.26	1.22 ± 0.27
z_X	$(1.7 \pm 1.4)10^{-3}$	$(2.7 \pm 2.2)10^{-4}$	$(1.6 \pm 1.5)10^{-3}$	$(3.0 \pm 2.1)10^{-3}$	$(6.0 \pm 5.8)10^{-4}$
y_Z	$(6.4 \pm 2.8)10{-}2$	$(2.0 \pm 0.9)10^{-2}$	$(6.1 \pm 2.5)10^{-2}$	$(9.0 \pm 3.8)10^{-2}$	$(4.6 \pm 2.4)10^{-2}$
x_Z	0.13 ± 0.05	0.11 ± 0.05	0.14 ± 0.05	0.14 ± 0.04	0.13 ± 0.05
x_Y	$(5.7 \pm 4.5)10^{-3}$	$(1.8 \pm 1.3)10^{-3}$	$(4.8 \pm 3.7)10^{-3}$	$(12.4 \pm 8)10^{-3}$	$(11 \pm 9)10^{-3}$
n	2.70 ± 0.15	2.44 ± 0.07	2.67 ± 0.14	2.42 ± 2.74	2.70 ± 0.16
σ	106 ± 44	135 ± 39	120 ± 42	114 ± 42	120 ± 40
S_{DC}	0.13 ± 0.13	0.22 ± 0.01	0.024 ± 25	0.132 ± 0.023	0.136 ± 0.023
d	0.33 ± 0.04	0.22 ± 0.01	0.32 ± 0.03	0.088 ± 0.023	0.359 ± 0.047

Table S.2: Related to STAR Methods. Inferred mean and standard deviation of the sampled parameter distribution for generations 8,9 and 10 (300 points). d^* is a second realisation of the inference using d to check the robustness of the optimisation. $d^{(13)}$ corresponds to generations 13, 14 and 15 for distance d. Alternative score functions d_1 and d_2 return similar parameter relationships. Parameter n is the varying Hill exponent corresponding to each score distance ($n = n_{zx}$ for d and d_1, and $n = n_{yz}$ for d_2), the rest of Hill exponents are fixed to 2).
