# Supplementary material for: Combining a Toggle Switch and a Repressilator within the AC-DC Circuit Generates Distinct Dynamical Behaviors
Source: Cell Syst. 2018 Apr 25;6(4):521–530.e3. doi: 10.1016/j.cels.2018.02.008 (PMC5929911; doi:10.1016/j.cels.2018.02.008)
Supplement: Document S2. Article plus Supplemental Information [file mmc11.pdf]

Cell Systems

Math | Bio

Combining a Toggle Switch and a Repressilator within the AC-DC Circuit Generates Distinct Dynamical Behaviors

Graphical Abstract

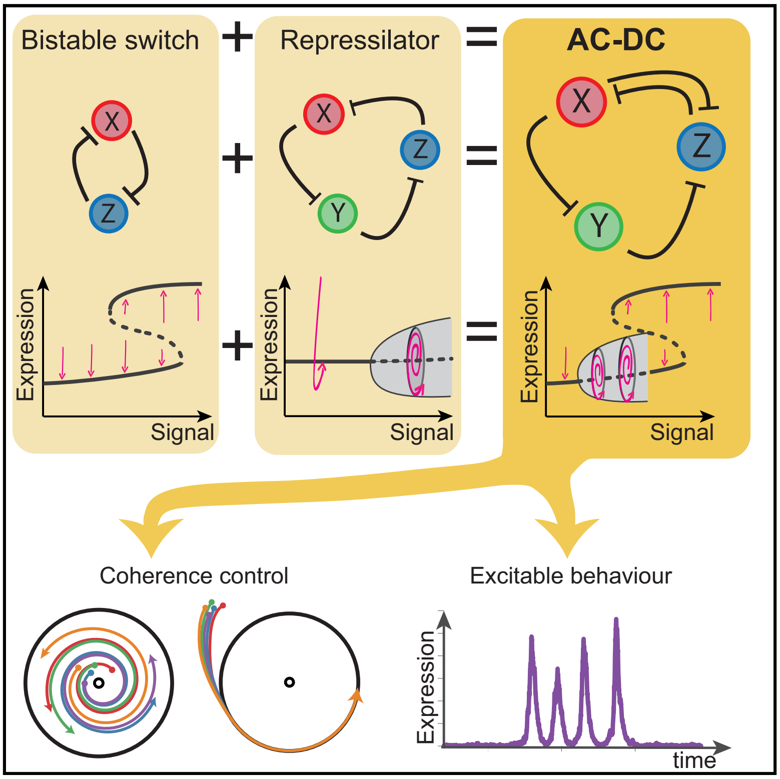

Authors

Ruben Perez-Carrasco, Chris P. Barnes, Yolanda Schaerli, Mark Isalan, James Briscoe, Karen M. Page

Correspondence

r.carrasco@ucl.ac.uk

In Brief

The AC-DC circuit, formed by the combination of a repressilator and a toggle switch, is explored in detail using dynamical systems theory and stochastic simulations. These analyses reveal that the coexistence of oscillatory and stable gene expression gives rise to novel dynamical behaviors such as control of oscillation coherence and spatial signal propagation.

Highlights

- The AC-DC circuit shows robust coexistence between oscillatory and steady expression
- The circuit allows control over the coherence of oscillations in a cell population
- The circuit shows excitable properties, allowing the spatial propagation of signals
- These suggest its prominence in development and its potential in synthetic biology

Perez-Carrasco et al., 2018, Cell Systems 6, 521–530
April 25, 2018 © 2018 The Author(s). Published by Elsevier Inc.
https://doi.org/10.1016/j.cels.2018.02.008

Cell Systems
Math | Bio

OPEN ACCESS
CellPress

Combining a Toggle Switch and a Repressilator within the AC-DC Circuit Generates Distinct Dynamical Behaviors

Ruben Perez-Carrasco,[1,7,*] Chris P. Barnes,[2,3] Yolanda Schaerli,[4] Mark Isalan,[5] James Briscoe,[6] and Karen M. Page[1]
[1]Department of Mathematics, University College London, Gower Street, WC1E 6BT London, UK
[2]Department of Cell and Developmental Biology, University College London, Gower Street, WC1E 6BT London, UK
[3]Department of Genetics, Evolution and Environment, University College London, Gower Street, WC1E 6BT London, UK
[4]Department of Fundamental Microbiology, University of Lausanne, Biophore Building, 1015 Lausanne, Switzerland
[5]Department of Life Sciences, Imperial College London, SW7 2AZ London, UK
[6]The Francis Crick Institute, 1 Midland Road, NW1 1AT London, UK
[7]Lead Contact
*Correspondence: r.carrasco@ucl.ac.uk
https://doi.org/10.1016/j.cels.2018.02.008

SUMMARY

Although the structure of a genetically encoded regulatory circuit is an important determinant of its function, the relationship between circuit topology and the dynamical behaviors it can exhibit is not well understood. Here, we explore the range of behaviors available to the AC-DC circuit. This circuit consists of three genes connected as a combination of a toggle switch and a repressilator. Using dynamical systems theory, we show that the AC-DC circuit exhibits both oscillations and bistability within the same region of parameter space; this generates emergent behaviors not available to either the toggle switch or the repressilator alone. The AC-DC circuit can switch on oscillations via two distinct mechanisms, one of which induces coherence into ensembles of oscillators. In addition, we show that in the presence of noise, the AC-DC circuit can behave as an excitable system capable of spatial signal propagation or coherence resonance. Together, these results demonstrate how combinations of simple motifs can exhibit multiple complex behaviors.

INTRODUCTION

Genetic circuits regulate biological functions in contexts that range from embryonic development to tissue homeostasis (Davidson, 2010). Accordingly, the analysis of the repertoire of functions performed by genetic circuits is central to systems biology. In some cases there is a direct relationship between the structure and operation of a circuit, such that the function—the dynamical behavior—of a circuit is evident from its topology. This has led to the classification of motifs or subnetworks based on topology and motivated the design and fabrication of artificial circuits with functions that include toggle switches, band-pass filters, memory devices, logic gates, and oscillators (Gardner et al., 2000; Elowitz and Leibler, 2000; Basu et al., 2005; Sohka et al., 2009; Ajo-Franklin et al., 2007; Siuti et al., 2013). Engineered versions of these circuits have been used to perform computation, screen for drugs, and detect and treat diseases (Daniel et al., 2013; Rubens et al., 2016; Xie et al., 2016).

Nevertheless, there is not always a one-to-one correspondence between topology and behavior. This is apparent from the analysis of even small circuits. In these cases, a small modification to such a circuit, for example the change in strength of interactions between components, leads to a qualitative change in the behavior of the circuit (Jia et al., 2017; Del Vecchio et al., 2008; Jayanthi et al., 2013; Tan et al., 2009; Prindle et al., 2014; Ingram et al., 2006). Far from being a nuisance, this has led to the concept of multifunctionality (Jiménez et al., 2017; Purcell et al., 2011)—circuits capable of qualitatively different outputs in a reduced parameter range. This poses the challenge of defining and predicting circuit behavior and emphasizes the importance of understanding the mapping between the topology of a genetic network and its dynamical behavior.

Identifying the minimal parameter changes necessary to elicit alternative behaviors from a multifunctional circuit provides insight into changes in behavior during gene network evolution and could be exploited for the engineering of circuits for synthetic biology tasks. Several studies have shed light on this problem through extensive numerical and experimental exploration of small networks targeting a specific function (Cotterell and Sharpe, 2010; Woods et al., 2016; Jiménez et al., 2017; Otero-Muras and Banga, 2016; Espinar et al., 2013; Schaerli et al., 2014). Insight from such studies is often obtained after the analysis and classification of the successful topologies in terms of the landscape of the corresponding dynamical system—sometimes called the geometrical landscape. The use of this dynamical landscape is key to revealing how different behaviors emerge, contributing to a better understanding of the mapping of topology to function (Jia et al., 2017; Strelkowa and Barahona, 2010; Süel et al., 2006; Jaeger and Monk, 2014; Verd et al., 2014).

Distilling minimal easy-to-engineer networks capable of specific functions is of paramount importance for engineering circuits for synthetic biology tasks (Schaerli et al., 2014; Purcell

This is an open access article under the CC BY license (http://creativecommons.org/licenses/by/4.0/).

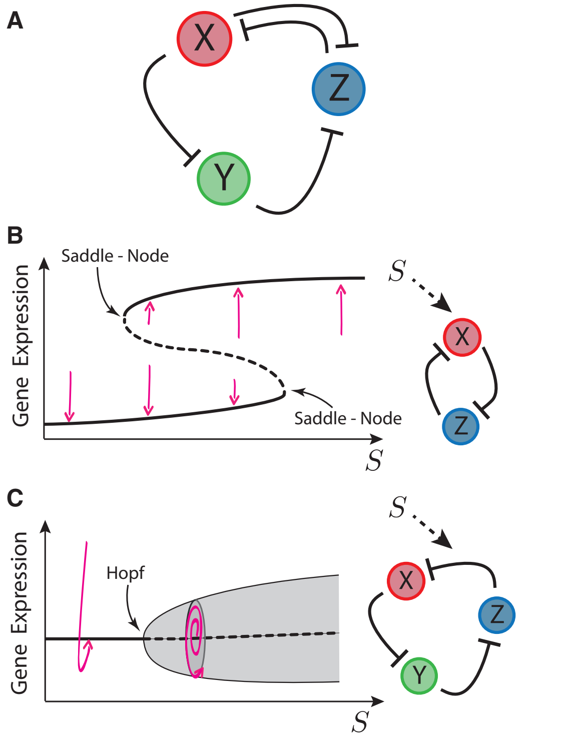

Figure 1. The AC-DC Circuit Is the Combination of a Bistable Switch and a Repressilator

(A) AC-DC regulatory circuit.

(B) Bifurcation diagram and network diagram for the bistable switch controlled by a signal *S*. The two saddle-node bifurcations position the stability range for the two stable solutions (black solid lines). There is bistability for intermediate signals, where both stable solutions are separated by an unstable steady state (dashed line). Transient trajectories (pink arrows) are sketched showing the dynamical effect of the steady states.

(C) Bifurcation diagram and network diagram for the repressilator under a change of parameters controlled with an external signal *S*. The change in behavior is a Hopf bifurcation where a stable spiral (damped oscillations) (thick black solid line) becomes unstable (dashed line) giving rise to stable oscillations (shaded zone). After the bifurcation, the amplitude of the stable oscillations (delimited by thin solid lines) grows with the signal. The two possible oscillatory transients are sketched (pink arrows).

et al., 2010; Chau et al., 2012). Cellular resources are scarce and implementing complex behaviors within cells requires an efficient and judicious use of these (Carrera et al., 2011; Mather et al., 2013; Cookson et al., 2011). Metabolic load affects gene expression through growth-dependent effects (Klumpp et al., 2009; Scott et al., 2010; Cardinale et al., 2013), and its reduction has become a major design objective (Ceroni et al., 2015; Borkowski et al., 2016). Minimal multifunctional circuits might offer a potential route to this goal. It is therefore important to find and understand the behaviors and emergent properties that can be encoded in a reduced gene circuitry. Theoretical and computational analyses have revealed that merely combining modules with different functions does not necessarily lead to additive outcomes. Conversely, in many cases, topologies capable of multifunctional behavior cannot be explained simply as the overlap of two or more submodules (Jiménez et al., 2017). A deeper understanding of the dynamics of multifunctional circuits is needed. As there is ample evidence that real biological systems exploit multifunctionality (Verd, 2016), designing and investigating such circuits is likely to shed light on biological processes (Mathur et al., 2017).

One attractive candidate for studying the coexistence and emergence of behavior in a multifunctional minimal network is the alternate current (AC)-direct current (DC) circuit (Panovska-Griffiths et al., 2013; Balaskas et al., 2012). Composed of two well-known subnetworks, the repressilator (Elowitz and Leibler, 2000; Purcell et al., 2010) and the toggle switch (Sokolowski et al., 2012; Gardner et al., 2000) (Figure 1A), the AC-DC circuit takes its name by analogy to AC and DC, since it is capable of generating oscillatory (AC) and multistable (DC) behavior (Panovska-Griffiths et al., 2013). The AC-DC circuit was originally observed in the patterning of progenitors in the vertebrate neural tube (Balaskas et al., 2012), where it is proposed to exhibit the DC behavior. Theoretical analysis revealed the potential for this circuit to generate oscillations inside a spatial pattern (Panovska-Griffiths et al., 2013) and the ability of the circuit topology to show stochastic switching between oscillations and steady-state expression (Li et al., 2012). Furthermore, Jaeger and colleagues have proposed that the gap gene system, which patterns the anterior-posterior axis of the *Drosophila melanogaster* embryo, is composed of three linked AC-DC circuits, two of which operate in the DC regime and one in the oscillatory, AC, mode (Verd, 2016).

The two subcomponents of the AC-DC circuit, the toggle switch and the repressilator, have been intensively studied, separately. Toggle switches consist of the cross-repression between the determinants of different cellular states and result in a definite choice between two outcomes. When controlled by an external signal, the toggle switch is able to produce a sharp transition between the steady states at a precise signal level (Figure 1B) (Sokolowski et al., 2012; Gardner et al., 2000). From a dynamical systems point of view, the sharp switch-like transition is the result of two saddle-node bifurcations. Each of these is characterized by the abrupt appearance of a stable and unstable steady expression state for a small change in the input signal. A consequence of this dynamical scenario is that, for a range of values of signal, both states are available and the expression state is determined by the initial gene expression. In addition, in the presence of noise, there is the possibility of switching between the stable states (Song et al., 2010; Tian and Burrage, 2006; Perez-Carrasco et al., 2016; Frigola et al., 2012).

The second component of the AC-DC circuit, the repressilator, comprises the sequential repression of three genes. In contrast to the toggle switch this provides a negative feedback loop that promotes stable oscillations. The amplitude and period of these depend on the parameters of the system (Elowitz and Leibler, 2000; Purcell et al., 2010). Changes in these parameters can lead to the disappearance of the oscillations through a Hopf bifurcation, in which the orbit in the expression space (limit cycle) shrinks, giving rise to a steady expression state. Hence coupling key parameters to an external signal can result in the repressilator becoming a switchable genetic oscillator, a property that has been extensively computationally explored (Buzzi and Llibre, 2015; Buşe et al., 2009; Purcell et al., 2010).

In this manuscript we characterize the functions of the AC-DC circuit by analyzing the phase portrait of the dynamical system.

We find that oscillations and stable expression can coexist in a large region of the parameter space, and we explore the implications of this coexistence. This reveals emergent behaviors not available to the repressilator or the toggle switch individually, which allow the circuit to be used to establish coherent or incoherent oscillations. In addition, we demonstrate that, with the addition of noise, the AC-DC circuit functions as an excitable system capable of coherence resonance and spatial signal propagation.

RESULTS

The Model

The expression dynamics of the AC-DC circuit can be described by taking into account the production and degradation of each gene (*X*, *Y*, and *Z*), where transcription processes are assumed to be faster than translation (Panovska-Griffiths et al., 2013). The production rate of each gene is regulated by the genetic interactions in the network and the inductive signal (*S*) that controls the behavior of the network and activates genes *X* and *Y*,

$$\dot{X} = \frac{\alpha_X + \beta_X S}{1 + S + (Z/z_X)^{n_{zx}}} - X,$$
$$\dot{Y} = \frac{\alpha_Y + \beta_Y S}{1 + S + (X/x_Y)^{n_{xy}}} - \delta_Y Y, \qquad \text{(Equation 1)}$$
$$\dot{Z} = \frac{1}{1 + (X/x_Z)^{n_{xz}} + (Y/y_Z)^{n_{yz}}} - \delta_Z Z.$$

Here all the variables and parameters are non-dimensional (see STAR Methods). The non-dimensional basal production rates α_X and α_Y are relative to the basal production rate of gene *Z*. The signal induction is controlled by parameters β_X and β_Y, while the strength and shape of the repressions are controlled by the non-dimensional factors z_X, x_Y, x_Z, and y_Z and the exponents n_{zx}, n_{xy}, n_{xz}, and n_{yz}. Finally, the rates δ_Z and δ_Y are the relative degradation rates to the degradation of gene *X*. Similarly, the time is measured in units of time of the degradation rate of protein *X*.

The use of non-dimensional parameters allows for the study of the minimal independent set of parameters required to define the possible different dynamics of the circuit, maximizing the information obtained for any parameter fitting of the model. In the present case we are interested in finding a global behavior of the AC-DC circuit without overfitting. For this reason we performed a minimal parameter exploration looking for behaviors that exhibit a transition from non-oscillatory to oscillatory behavior through a change in the signal. In addition, we required the Hill exponents to be as low as possible to avoid numerical artifacts due to high nonlinearities. This would also ensure a set of parameters achievable in synthetic circuits.

The parameter exploration was performed using approximate Bayesian computation (Liepe et al., 2010; Liepe et al., 2014) and gave as a result the distribution of parameters necessary for observing a tunable oscillator in the AC-DC circuit. The resulting parameters (Table S1) return a consistent relationship between the parameters for different target optimizations (see STAR Methods). Namely, the basal production rate of the different genes has a marked hierarchy with *Z* being the largest, followed by genes *X* and *Y*. By contrast, both signal activation strengths are similar $(\beta_X \simeq \beta_Y)$. In addition, the strongest repression is that of *X* from *Z*, while the weakest is its reciprocal, from *X* to *Z*. The repressions unique to the repressilator, x_y and y_z, are in between these values. All the optimizations returned a difference of at least one order of magnitude between the different repression magnitudes ($z_x < x_y < y_z < x_z$), with clear correlations between them. Notably, a similar degradation rate was observed for all three proteins $\delta_Y \simeq \delta_Z \simeq 1$. Finally, no oscillations were found when Hill exponents $n = 2$ were used, but a small increase of only one of the Hill exponents provided sufficient non-linearity to observe oscillations.

In addition to the deterministic model, it is also informative to test the behavior of the AC-DC circuit subjected to molecular intrinsic noise derived from the deterministic Equations (1) as chemical Langevin equations (Gillespie, 2000) (see STAR Methods). The inclusion of intrinsic noise has two purposes, it shows the robustness of some of the functionalities to fluctuations, while revealing new phenomena not available in a deterministic scenario.

The AC-DC Circuit Shows Bistability between Oscillations and Steady Expression

Analysis of the bifurcation diagram of the circuit reveals a mixture of the bistability from the toggle switch and the oscillatory behavior of the repressilator, in a similar way to other circuits comprising an incoherent feedback (Pfeuty and Kaneko, 2009). The Hopf bifurcation by which the oscillations arise in the repressilator transforms one of the stable states of the bistable switch into an oscillatory state that can coexist for a certain signal range with the other stable state (Figure 2; Movies S1, S2, and S3). Hence, for a given value of signal *S* both behaviors (oscillatory or stable expression) are possible. The chosen state will depend on the history of the system *S*, i.e., the system displays hysteresis. This behavior was present in 80% of the optimized parameter sets even though the parameter search optimization did not score for any kind of bistability. This suggests that it is a robust behavior arising from the network topology.

Overall, examining the bifurcation diagram shows that the behavior consisted of four different dynamical regimes for different signal ranges. For low values of the signal, there is only one possible steady state with low expression of gene *X*, resulting from a low activation of the promoters *X* and *Y* by the signal. As the signal increases, the system starts to oscillate through a Hopf bifurcation, with oscillations of small amplitude that increases with the amount of signal. For larger values of the signal a new stable state with high expression of *X* becomes available through a saddle-node bifurcation. This new state appears away from the limit cycle (steady oscillatory trajectory) without affecting it, giving rise to the bistable regime between oscillations and constant expression.

For large values of the signal the oscillations disappear. The bifurcation analysis indicated that two different mechanisms could be responsible for this. On the one hand, a Hopf bifurcation may arise collapsing the limit cycle before the second saddle-node occurs (Figure 2). On the other hand, the oscillatory state can collide with the unstable steady state produced by the saddle-node bifurcation, resulting in a homoclinic bifurcation, as previously observed by (Li et al., 2012). This gives rise to a

OPEN ACCESS
CellPress

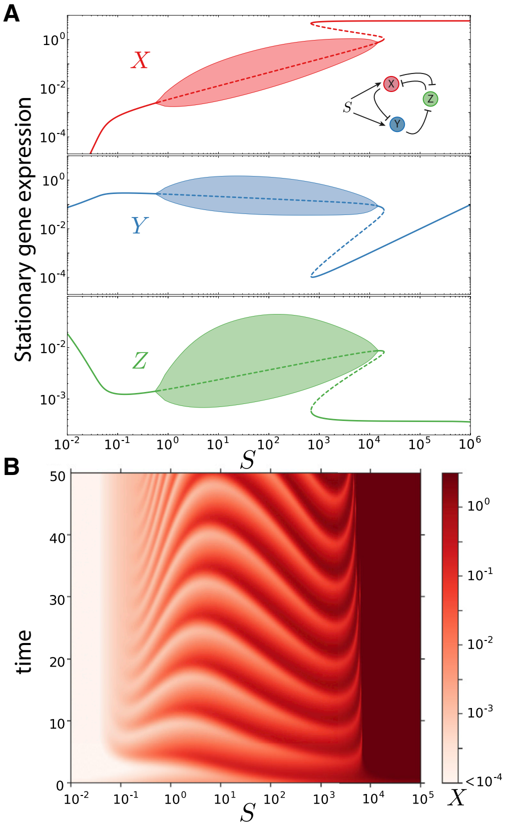

Figure 2. Dynamical Regimes of the AC-DC Circuit

(A) Stability diagram showing the available steady states of each gene for different values of the signal S using the parameters of Table S1. Thick solid lines show stable steady states, dashed lines show unstable states. Shaded areas show the range of expression of oscillatory states, which are delimited by thin solid lines. The bifurcation diagram was obtained using integration and continuation techniques (Clewley, 2012). See also Figure S1.

(B) Expression of gene X in time for different signal levels exhibiting three different dynamical regions. Initial condition is $X = 0$, $Y = 0$, $Z = 0$.

regime in which, even though the oscillatory state is not stable, it readily generates oscillatory transients toward the steady state (Figure S1). We will consider the first case for the rest of this study; nevertheless, all the behaviors described herein are independent of the mechanism by which the oscillations disappear.

An additional regime may appear in which two constant expression steady states coexist for a wide range of signal, as expected from any bistable switch (Figure 1B). The availability of this regime depends on the location of the oscillatory region with respect to the two saddle-node bifurcations (Figure S1). Since our parameter exploration maximizes oscillatory behavior, this dynamical regime was not frequent and will not be considered in the rest of the study, which focuses instead on the coexistence between sustained oscillations and constant expression steady states.

Coherent or Incoherent Oscillations

One way of inducing oscillations through a change in signal is to increase the signal from low levels to a level above the Hopf bifurcation threshold (ΔS_1). In addition, the coexistence of oscillations with saddle-node bifurcations allows for an alternative way to initiate oscillations. Starting from the stable expression state achieved at high signal levels, the oscillatory state can be reached by reducing the signal below the saddle-node bifurcation (ΔS_2) (see Figure 3). Whereas in the first case a small limit cycle arises around the initial steady state, in the second case a large amplitude limit cycle is already present within the dynamical landscape when the bifurcation takes place. These differences result in different dynamical transients toward the oscillatory state.

To test these differences we performed simulations of the stochastic model starting at low or high signal and ending at the same intermediate signal value. Results show that in the first scenario—increasing signal from a low value—gives rise to asynchronous oscillations in a population of cells. Small differences in the initial phase are amplified over time. By contrast, the second scenario—decreasing signal from a high level—induces coherent oscillations in the face of noise (Figure 3; Movies S4 and S5).

This difference in behavior is a consequence of the different initial gene expression states in relation to oscillatory spiral center. In a Hopf bifurcation, oscillations arise through an attracting spiral losing its stability and becoming a repulsing spiral. Hence, oscillations originating from a Hopf bifurcation start their transient close to the unstable spiral center, and a small variation in the initial condition can lead to a substantial difference in the final oscillation phase. Small initial differences are amplified, resulting in lack of coherence of oscillations for a population of cells undergoing the bifurcation. By contrast, cells passing through the saddle-node bifurcation toward the limit cycle do so at expression levels that are far from those associated with the attracting oscillatory regime. Consequently, they have the same initial phase, and stochastic trajectories are canalized together toward the oscillatory state. In this way the AC-DC circuit, for a single set of parameters, offers the possibility to establish either coherent or incoherent oscillations in a population by choosing the appropriate signal transient. Specifically, the second mechanism is not available in the original repressilator since it requires the bistability provided by the toggle switch.

In addition to the synchrony of response, it is important to note that the saddle-node bifurcation also allows the rapid establishment of constant amplitude oscillation after the signal is reduced. Thus, the AC-DC system offers a fast mechanism to turn on and off the oscillations, which is not a feature of the repressilator. Previous studies propose robust switching exploiting quasi-stable oscillatory transients in repressilators with an even number of repressions (Strelkowa and Barahona, 2010). In contrast the AC-DC circuit exhibits the benefits of both systems, the robustness of oscillating with a stable limit cycle, and the fast switchability, in this case provided by a bifurcation occurring far from the central unstable spiral.

The AC-DC Circuit Shows Excitability

The long-term behavior of the deterministic system in the bistable zone of the AC-DC circuit is determined by the initial conditions of the system. The set of initial conditions that are attracted to each of the two possible stable states are their respective basins of attraction. Intrinsic fluctuations in the expression levels allow the system to explore the basin of

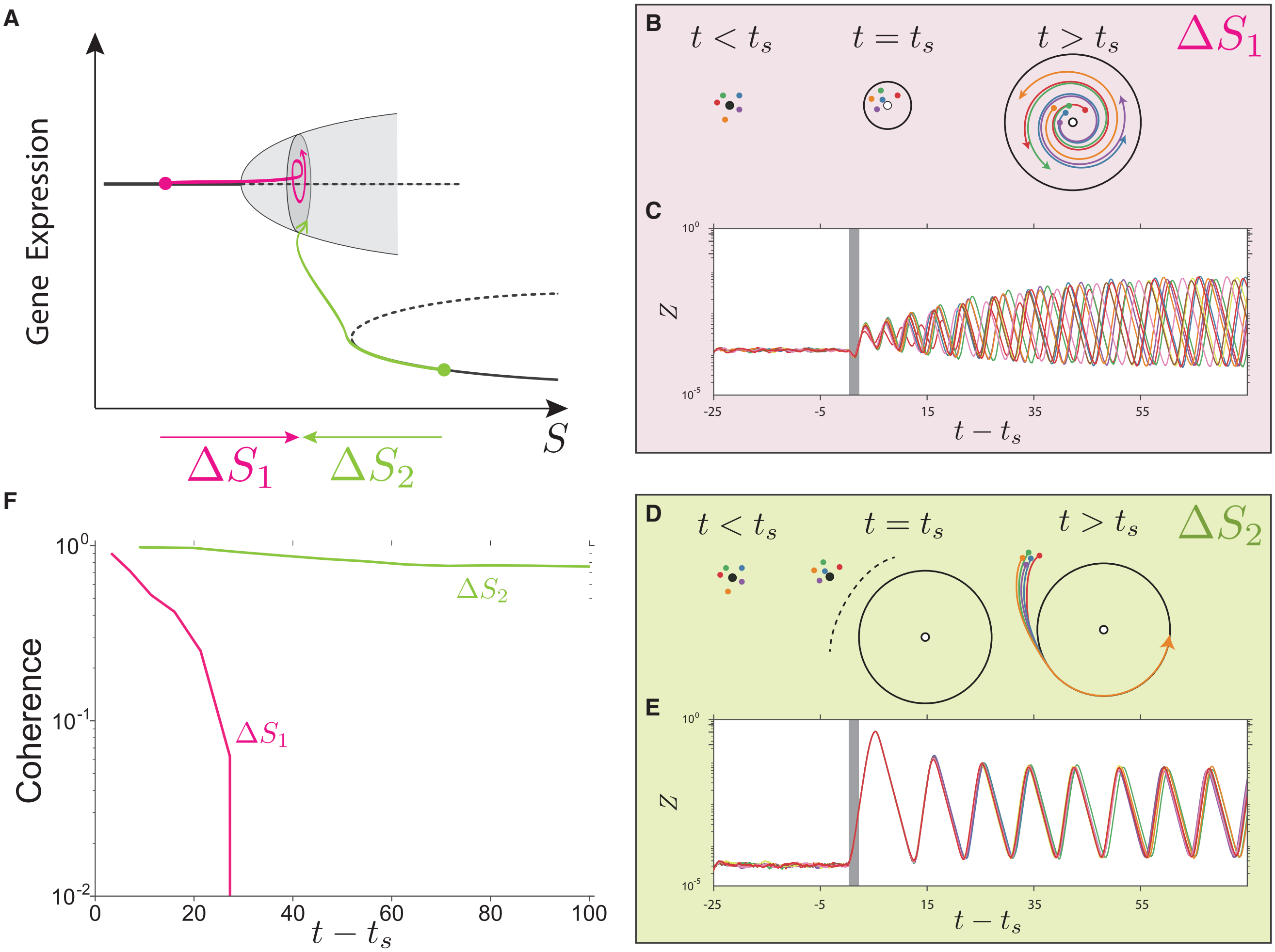

Figure 3. AC-DC Circuit Allows the Control of Oscillation Coherence between Different Cells

(A) Schematic showing the two possible transitions toward the limit cycle.

(B) Oscillations arising through the Hopf bifurcation (ΔS_1) are incoherent. Diagrams show steady states and transients in the genetic expression plane. Initially, there is only one stable state (solid black circle) of constant expression, the genetic expression of different cells (colored circles) is determined by this stable state. After the signal is increased at $t = t_s$, the steady state becomes unstable giving place to an unstable spiral center (solid white circle) and a stable limit cycle (black circumference). The resulting dynamical behavior for the different cells (colored arrows) follows a spiral transient toward the limit cycle.

(C) Simulations of ΔS_1 show the appearance of oscillations that lose their coherence by increasing the signal from $S = 0.1$ to $S = 100$ at $t = t_s$ for $\Delta t = 2$ (gray shaded area), $\Omega = 10^6$.

(D) Oscillations through the saddle-node bifurcation (ΔS_2) are coherent. Diagrams show steady states and transients in the genetic expression plane. Initially, expression of cells (colored circles) are found in a stable state (solid black circle) of constant expression. After the signal is increased at $t = t_s$, the steady state disappears from the plane (solid white circle) and the only attractor available is the limit cycle (black circumference), which imposes a fast expression transient toward the stable oscillations (colored lines).

(E) Simulations of ΔS_2 show the appearance of coherent oscillations by decreasing the signal from $S = 105$ to $S = 100$ at $t = t_s$ for $\Delta t = 2$ (gray shaded area), $\Omega = 10^6$.

(F) Comparison of the decrease in coherence in time for both signal histories ΔS_1 and ΔS_2 measured as $(\sigma_{max} - \sigma(t))/\sigma_{max}$, where σ is the SD of the phase of the oscillations for 20 simulations of each mechanism and σ_{max} is the SD corresponding to completely incoherent oscillations.

attraction, or even to cross between basins, resulting in noise-induced transitions between different cellular states (Song et al., 2010; Jia et al., 2017). In the case of the AC-DC circuit, the switching capabilities between oscillations and constant expression by intrinsic noise was analyzed by (Li et al., 2012), revealing that the frequency of switching depends on the geometry of the basin and the level of intrinsic noise. Here we explore possible effects and functionalities of this transition by altering the signal S and intrinsic noise levels with the system volume parameter Ω (see STAR Methods)(Gillespie, 2000; Perez-Carrasco et al., 2016).

Switching between states is not equally probable for all levels of gene expression (de la Cruz et al., 2017). In particular, once the system jumps into the oscillatory state, at least one excursion around the limit cycle is required before it can return to the constant expression state (Figure 4). Such an excursion results in the amplification of a transient fluctuation. In addition, this excursion entails a refractory period during which the system

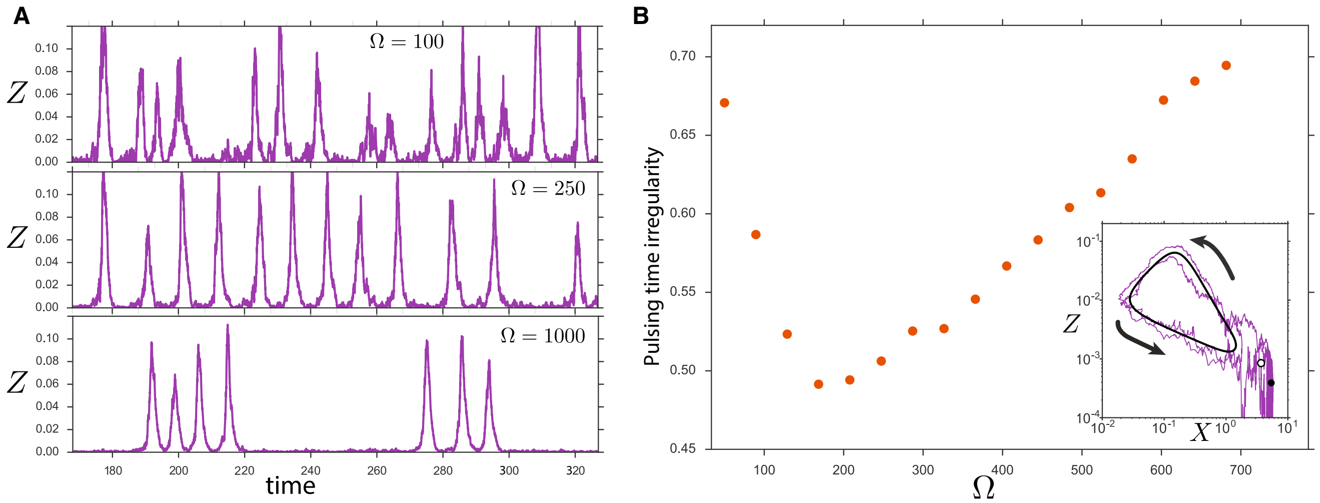

Figure 4. AC-DC Circuit Shows Coherence Resonance

(A) Pulses of gene expression for three different noise levels. The most regular pulsing occurs for intermediate noise ($\Omega \simeq 250$). Simulations performed with $S = 700$.

(B) Irregularity of times between pulses as a function of noise intensity. The irregularity for each value of Ω is measured as the coefficient of variation of the times between expression peaks of gene Z for trajectories during $\Delta t = 50{,}000$. This illustrates minimal irregularity for intermediate Ω. Inset: Expression levels of genes X and Z during an activation that lasts for two pulses. Stochastic fluctuations drive the system far from the steady state (black circle) past the unstable steady state (white circle), driving the system around the limit cycle (black orbit). Arrows show the direction of the limit cycle. $\Omega = 1{,}000$, $S = 700$.

cannot be triggered again until the full cycle is finished. This pulsatile behavior reveals the excitable nature of the AC-DC circuit. Excitability has been found in other genetic systems where expression pulses have been suggested to be beneficial for the biology of the cell (Süel et al., 2006; Levine et al., 2013). In many cases the excitability arises from incoherent feedbacks (Pfeuty and Kaneko, 2009). This is the case for the AC-DC circuit where the incoherent feedback is a consequence of the superposition of its two subcircuits—the bistable switch (positive feedback) and the repressilator (negative feedback).

The frequency of the pulses depends on the noise intensity and value of the signal. In situations with low noise, the system can be trapped in the oscillatory state for more than one period (Figure 4), leading to the spike trains studied by (Li et al., 2012), which are similar to the spike trains observed in neuronal activity (Lindner et al., 2004). By contrast, if intrinsic noise is increased, the probability of observing isolated spike trains decreases. The increase in intrinsic noise results in a greater chance of exiting the limit cycle, but also a greater probability of inducing another excitation after each refractory period. This results in an increased regularity of the pulses as noise intensity augments. However, with large levels of intrinsic noise the quality of the oscillations and refractory period is disrupted, leading to a decrease in the regularity of the pulsing (Figure 4). Hence there is an optimal constructive level of noise for which the spikes become more regular. This effect, which is known as coherence resonance, is common in excitable systems and provides a way to exploit intrinsic noise for signal detection (Lindner et al., 2004; Bates et al., 2014).

The excitable nature of the AC-DC circuit might also be relevant to other functions, such as signal propagation in a tissue. A cell signaling to neighboring cells can be excited to undergo a pulse that will, in turn, excite neighboring cells and so on. The intensity and transient and refractory periods of the pulse not only contribute to the excitation of neighboring cells but also inhibit the reactivation of the recently excited cells, resulting in a spatially propagating pulse over the tissue. To test this possibility, we performed a series of numerical assays in a simulated tissue where one or more of the proteins forming the AC-DC circuit diffuse between cells. To initiate the system, bistable cells are set in the constant stable expression state. In this scenario, the induced excitation of one cell leads to a propagating front in which cells are excited sequentially, returning afterward to the initial constant expression state (Figure 5; Movie S6). During this period of time, the transient expression along the limit cycle is high enough to deliver the pulse to the neighboring cells. The width and velocity of the propagating front can be controlled with the noise intensity or the diffusion coefficient, as well as the signaling mechanism between cells. In addition, for a high enough level of noise, spontaneous propagation waves can also occur, as well as dynamical patterning of the system (see Movies S6, S7, S8, and S9). Similar results would be expected with more elaborate signaling pathways in which transmembrane receptors are involved in the transmission of the signal (Mathur et al., 2017; Jiménez et al., 2017; Formosa-Jordan et al., 2012).

DISCUSSION

We have explored the behavior of the multifunctional AC-DC circuit, showing that the coexistence between bistability and oscillations elicit novel dynamics that are unavailable to either of its constituent parts. These provide a mechanism to rapidly switch between a rhythmic and steady expression regime and suggests a way in which coherent oscillations can be induced into an ensemble of otherwise noisy oscillators. In addition, we demonstrate the excitable dynamics of the system that result in the potential for coherence resonance and spatial signal

OPEN
ACCESS
CellPress

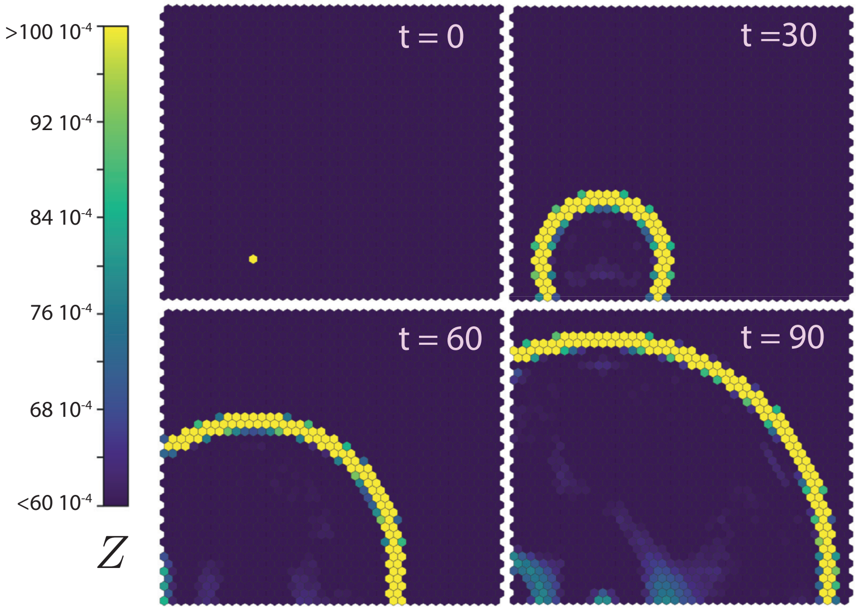

Figure 5. AC-DC Pulse Can Be Used as a Spatial Signal Propagation in an Array of Cells Containing the AC-DC Circuit

Proteins X, Y, and Z can diffuse intercellularly. Cells are under signal S = 1,000 (bistable regime) and initially set at the constant expression steady state except one cell that is perturbed away from the steady state. This perturbation initiates a wave that propagates through the field. $\Omega = 10^5$, $D = 0.1$ (see more details in the Supplemental Information).

propagation. Notably, these behaviors are accessible for the same range of parameters, supporting the idea that the circuit represents a versatile genetically encoded network well suited for a range of functions.

Although not apparent from the structure of the network, the various functions of the AC-DC circuit become evident from an inspection of the geometry of its dynamical landscape. As has been shown in previous studies, the position and nature of the attractors of the system provide substantially greater insight than a simple analysis of the topology of the network (Jiménez et al., 2017; Cotterell and Sharpe, 2010; Süel et al., 2006; Strelkowa and Barahona, 2010; Jia et al., 2017; Jaeger and Monk, 2014; Verd et al., 2014; Pfeuty and Kaneko, 2009). For the AC-DC circuit, the shape of the dynamical landscape is created by the combination of saddle-node bifurcation arising from the toggle switch and the Hopf bifurcation of the repressilator. Moreover, transitions such as the sudden destabilization of oscillations through the homoclinic bifurcation, can be anticipated from examining the structure of the dynamical landscape. This highlights the application and importance of dynamical systems tools for identifying and explaining the behavior of even relatively simple circuits. It also raises the possibility that similar dynamical behaviors might be present in other circuits composed of incoherent feedbacks that share similar attractor landscapes (Pfeuty and Kaneko, 2009; Krishna et al., 2009).

Besides its potential applications in synthetic biology (see Box 1), the AC-DC circuit also offers insight into genetic circuits involved in tissue development. During vertebrate embryogenesis, coordinated gene expression oscillations—the segmentation clock—in the posterior cells of the body generate a rhythmic spatial pattern that subdivides the embryonic trunk into morphological segments (Hubaud and Pourquié, 2014). This involves a still poorly defined genetic oscillator within posterior cells. Notably, individual cells appear to behave as autonomous oscillators that, when isolated from the embryo, produce transient stochastic periods of oscillations (Webb et al., 2016). In addition, a recent experimental study has revealed the excitable nature of such oscillations (Hubaud et al., 2017). This behavior features the same properties found in the excitable regime of the AC-DC circuit in which a limit cycle and steady expression state coexist, suggesting that the AC-DC circuit could provide a model for the process. In a different tissue, the *Drosophila* blastoderm, the dynamics of three linked AC-DC gene circuits have been proposed to characterize the regulatory network that patterns the anterior-posterior axis (Verd, 2016). In this case, the dynamical transients of the AC-DC circuit are suggested to tune the position of the boundaries in time. Moreover, the presence of AC-DC dynamics in this gene network has been suggested later on to reconcile differences between short and long germband insects (Clark, 2017). In short germband insects, rhythmic expression of genes is associated with the gradual extension of the body axis. By contrast in long germband insects, such as *Drosophila*, the trunk is patterned simultaneously without cyclic expression of the patterning genes. The bistability between a stable steady state and a limit cycle and the possibilities to transition smoothly between regimes with a change of one parameter suggests a route for the evolutionary transition of the underlying gene-regulatory network (Verd, 2016). In this view, the multifunctionality of the AC-DC circuit contributes to the evolvability of the circuit and exemplifies how the competing demands of biological mechanisms to be both robust and adaptable can be satisfied. Accordingly, studies of genetically encoded circuits such as the AC-DC network provide insight into the design principles of regulatory mechanisms that characterize biology.

STAR★METHODS

Detailed methods are provided in the online version of this paper and include the following:

- KEY RESOURCES TABLE
- CONTACT FOR REAGENT AND RESOURCE SHARING
- METHOD DETAILS
  - Non-dimensional Equations
  - Parameter Fitting
  - Stochastic Expression
  - Cell Lattice Diffusion

Box 1. Applications of the AC-DC Circuit in Synthetic Biology

Of particular interest for the AC-DC circuit is the multistability and the ability to control switching between the different behaviors by changing a single external signal. The availability of wide regions of parameter space, in which these behaviors take place, makes the AC-DC circuit an attractive target for potential applications in synthetic biology. In particular, the problem of generating synchronized ensembles of oscillators is a challenge. Considerable efforts have been made to solve this problem either by engineering away noise or relying on quorum sensing (McMillen et al., 2002; Garcia-Ojalvo et al., 2004; Kobayashi et al., 2004; Kuznetsov et al., 2006; Danino et al., 2010; Nikolaev and Sontag, 2016; Potvin-Trottier et al., 2016) The AC-DC circuit offers a novel strategy that relies on the bistable oscillatory regime, producing a favorable robust dynamical transient toward the oscillations. Moreover, the circuit offers the possibility of exhibiting different degrees of coherence in response to a single triggering signal. This offers a new tool for synthetic biology to control the heterogeneity of gene expression in a population of cells.

Pulsatile excitations are also a property exploited in several biological situations. The behavior is reminiscent of bet-hedging strategies that have been proposed to optimize responses to external inputs or maximize the use of limited resources by controlling the time at which nutrient demanding physiological processes occur (Levine et al., 2013; Süel et al., 2006; Liu et al., 2017). From this perspective, the AC-DC circuit provides a mechanism to explore different excitable regimes by changing the external signal without the need to control the levels of intrinsic noise by altering the copy number or degradation rates of the system (Hilborn et al., 2012; Niederholtmeyer et al., 2015).

Moreover, the AC-DC circuit exhibits pulsatile properties. In other circuits the excitability arises from unstable excitable transients or through a subcritical Hopf bifurcation (Süel et al., 2006,; Hilborn et al., 2012). In the AC-DC circuit, the separation of the saddle-node bifurcation that initiates oscillations from the amplitude of the limit cycle allows for parameterizations in which both properties of the bistable region can be tuned independently to control the different features of the pulses. Similar distinctions are found in excitable systems, such as those associated with neuronal action potentials (Izhikevich, 2000; Lindner et al., 2004), raising the possibility of combining current advances in neuronal networks and excitable media with synthetic genetic circuits. In particular, the tunability of the excitable properties allows for signal propagation across a population of cells with different velocities and intensities that can be slower than the typical production and degradation rates of the molecular components of the circuit. In addition, it suggests the possibility of exploiting coherence resonance for signal detection. This property results when an increase in noise intensity produces an increased probability of crossing a critical threshold in excitable systems. In both neurological and manufactured systems this can be used to increase the signal-to-noise ratio and therefore to enhance the detection of weak signals (Lindner et al., 2004; Bates et al., 2014). The capacity of the AC-DC circuit for coherence resonance raises the possibility of exploiting this feature in the design of biosensing applications in synthetic biology (Van Der Meer and Belkin, 2010).

SUPPLEMENTAL INFORMATION

Supplemental Information includes three figures, two tables, and nine movies and can be found with this article online at https://doi.org/10.1016/j.cels.2018.02.008.

ACKNOWLEDGMENTS

We acknowledge Berta Verd for fruitful discussions and the editor Quincey Justman for insightful suggestions on the title and abstract of the manuscript. R.P.-C. and K.M.P. would like to acknowledge support from the Wellcome Trust (grant reference WT098325MA). C.P.B. is funded through a Wellcome Trust Research Career Development Fellowship (097319/Z/11/Z). M.I. is funded by a Wellcome Trust UK New Investigator Award (WT102944). Y.S. acknowledges support from the Swiss National Science Foundation (Ambizione program PZ00P3-148235). J.B. is supported through the Francis Crick Institute by funding from Cancer Research UK (FC001051), the UK Medical Research Council (FC001051), and the Wellcome Trust (FC001051 and WT098326MA).

AUTHOR CONTRIBUTIONS

R.P.-C. and K.M.P. designed the research. R.P.-C. and C.P.B. performed the research. R.P.-C., C.P.B., Y.S., M.I., J.B., and K.M.P. wrote the paper.

DECLARATION OF INTERESTS

The authors declare no competing interests.

Received: September 26, 2017
Revised: December 14, 2017
Accepted: February 13, 2018
Published: March 21, 2018

REFERENCES

Ajo-Franklin, C.M., Drubin, D.A., Eskin, J.A., Gee, E.P., Landgraf, D., Phillips, I., and Silver, P.A. (2007). Rational design of memory in eukaryotic cells service rational design of memory in eukaryotic cells. Genes Dev. *21*, 2271–2276.

Balaskas, N., Ribeiro, A., Panovska, J., Dessaud, E., Sasai, N., Page, K.M., Briscoe, J., and Ribes, V. (2012). Gene regulatory logic for reading the Sonic Hedgehog signaling gradient in the vertebrate neural tube. Cell *148*, 273–284.

Basu, S., Gerchman, Y., Collins, C.H., Arnold, F.H., and Weiss, R. (2005). A synthetic multicellular system for programmed pattern formation. Nature *434*, 1130–1134.

Bates, R., Blyuss, O., and Zaikin, A. (2014). Stochastic resonance in an intracellular genetic perceptron. Phys. Rev. E Stat. Nonlin. Soft Matter Phys. *89*, 032716.

Borkowski, O., Ceroni, F., Stan, G.B., and Ellis, T. (2016). Overloaded and stressed: whole-cell considerations for bacterial synthetic biology. Curr. Opin. Microbiol. *33*, 123–130.

Buzzi, C.A., and Llibre, J. (2015). Hopf bifurcation in the full repressilator equations. Math. Methods Appl. Sci. *38*, 1428–1436.

Buşe, O., Kuznetsov, A., and Pérez, R.A. (2009). Existence of limit cycles in the repressilator euqations. Int. J. Bifurc. Chaos *19*, 4097–4106.

Cardinale, S., Joachimiak, M.P., and Arkin, A.P. (2013). Effects of genetic variation on the *E. coli* host-circuit interface. Cell Rep. *4*, 231–237.

Carrera, J., Rodrigo, G., Singh, V., Kirov, B., and Jaramillo, A. (2011). Empirical model and in vivo characterization of the bacterial response to synthetic gene expression show that ribosome allocation limits growth rate. Biotechnol. J. *6*, 773–783.

Ceroni, F., Algar, R., Stan, G.B., and Ellis, T. (2015). Quantifying cellular capacity identifies gene expression designs with reduced burden. Nat. Methods *12*, 415–418.

Chau, A.H., Walter, J.M., Gerardin, J., Tang, C., and Lim, W.A. (2012). Designing synthetic regulatory networks capable of self-organizing cell polarization. Cell *151*, 320–332.

Clark, E. (2017). Dynamic patterning by the *Drosophila* pair-rule network reconciles long-germ and short-germ segmentation. PLoS Biol. *15*, e2002439.

Clewley, R. (2012). Hybrid models and biological model reduction with PyDSTool. PLoS Comput. Biol. *8*, e1002628.

Cookson, N.A., Mather, W.H., Danino, T., Mondragón-Palomino, O., Williams, R.J., Tsimring, L.S., and Hasty, J. (2011). Queueing up for enzymatic processing: correlated signaling through coupled degradation. Mol. Syst. Biol. *7*, 561.

Cotterell, J., and Sharpe, J. (2010). An atlas of gene regulatory networks reveals multiple three-gene mechanisms for interpreting morphogen gradients. Mol. Syst. Biol. *6*, 425.

Daniel, R., Rubens, J.R., Sarpeshkar, R., and Lu, T.K. (2013). Synthetic analog computation in living cells. Nature *497*, 619–623.

Danino, T., Mondragón-Palomino, O., Tsimring, L., and Hasty, J. (2010). A synchronized quorum of genetic clocks. Nature *463*, 326–330.

Davidson, E. (2010). The Regulatory Genome: Gene Regulatory Networks in Development and Evolution (Elsevier Science).

de la Cruz, R., Perez-Carrasco, R., Guerrero, P., Alarcon, T., and Page, K.M. (2017). Minimum Action Path theory reveals the details of stochastic biochemical transitions out of oscillatory cellular states. arXiv, arXiv1705.08683.

Del Vecchio, D., Ninfa, A.J., and Sontag, E.D. (2008). Modular cell biology: retroactivity and insulation. Mol. Syst. Biol. *4*, 161.

Elowitz, M.B., and Leibler, S. (2000). A synthetic oscillatory network of transcriptional regulators. Nature *403*, 335–338.

Espinar, L., Dies, M., Cagatay, T., Süel, G.M., and Garcia-Ojalvo, J. (2013). Circuit-level input integration in bacterial gene regulation. Proc. Natl. Acad. Sci. USA *110*, 7091–7096.

Formosa-Jordan, P., Ibanes, M., Ares, S., and Frade, J.M. (2012). Regulation of neuronal differentiation at the neurogenic wavefront. Development *139*, 2321–2329.

Frigola, D., Casanellas, L., Sancho, J.M., and Ibañes, M. (2012). Asymmetric stochastic switching driven by intrinsic molecular noise. PLoS One *7*, e31407.

Garcia-Ojalvo, J., Elowitz, M.B., and Strogatz, S.H. (2004). Modeling a synthetic multicellular clock: repressilators coupled by quorum sensing. Proc. Natl. Acad. Sci. USA *101*, 10955–10960.

Gardner, T.S., Cantor, C.R., and Collins, J.J. (2000). Construction of a genetic toggle switch in *Escherichia coli*. Nature *403*, 339–342.

Gillespie, D.T. (2000). The chemical Langevin equation. J. Chem. Phys. *113*, 297.

Hilborn, R.C., Brookshire, B., Mattingly, J., Purushotham, A., and Sharma, A. (2012). The transition between stochastic and deterministic behavior in an excitable gene circuit. PLoS One *7*, e34536.

Hubaud, A., and Pourquié, O. (2014). Signalling dynamics in vertebrate segmentation. Nat. Rev. Mol. Cell Biol. *15*, 709–721.

Hubaud, A., Regev, I., Mahadevan, L., and Pourquié, O. (2017). Excitable dynamics and Yap-dependent mechanical cues drive the segmentation clock. Cell *171*, 668–682.e11.

Ingram, P.J., Stumpf, M.P., and Stark, J. (2006). Network motifs: structure does not determine function. BMC Genomics *7*, 108.

Izhikevich, E.M. (2000). Neural excitability, spiking and bursting. Int. J. Bifurc. Chaos *10*, 1171–1266.

Jaeger, J., and Monk, N. (2014). Bioattractors: dynamical systems theory and the evolution of regulatory processes. J. Physiol. *592*, 2267–2281.

Jayanthi, S., Nilgiriwala, K.S., and Del Vecchio, D. (2013). Retroactivity controls the temporal dynamics of gene transcription. ACS Synth. Biol. *2*, 431–441.

Jia, D., Jolly, M.K., Harrison, W., Boareto, M., Ben-Jacob, E., and Levine, H. (2017). Operating principles of tristable circuits regulating cellular differentiation. Phys. Biol. *14*, 035007.

Jiménez, A., Cotterell, J., Munteanu, A., and Sharpe, J. (2017). A spectrum of modularity in multi-functional gene circuits. Mol. Syst. Biol. *13*, 925.

Klumpp, S., Zhang, Z., and Hwa, T. (2009). Growth rate-dependent global effects on gene expression in bacteria. Cell *139*, 1366–1375.

Kobayashi, H., Kaern, M., Araki, M., Chung, K., Gardner, T.S., Cantor, C.R., and Collins, J.J. (2004). Programmable cells: interfacing natural and engineered gene networks. Proc. Natl. Acad. Sci. USA *101*, 8414–8419.

Krishna, S., Semsey, S., and Jensen, M.H. (2009). Frustrated bistability as a means to engineer oscillations in biological systems. Phys. Biol. *6*, 036009.

Kuznetsov, A., Kaern, M., and Kopell, N. (2006). Synchrony in a population of hysteresis-based genetic oscillators. SIAM J. Appl. Math. *65*, 392–425.

Levine, J.H., Lin, Y., and Elowitz, M.B. (2013). Functional roles of pulsing in genetic circuits. Science *342*, 1193–1200.

Li, W., Krishna, S., Pigolotti, S., Mitarai, N., and Jensen, M.H. (2012). Switching between oscillations and homeostasis in competing negative and positive feedback motifs. J. Theor. Biol. *307*, 205–210.

Liepe, J., Barnes, C., Cule, E., Erguler, K., Kirk, P., Toni, T., and Stumpf, M.P. (2010). ABC-SysBio – approximate Bayesian computation in Python with GPU support. Bioinformatics *26*, 1797–1799.

Liepe, J., Kirk, P., Filippi, S., Toni, T., Barnes, C.P., and Stumpf, M.P. (2014). A framework for parameter estimation and model selection from experimental data in systems biology using approximate Bayesian computation. Nat. Protoc. *9*, 439–456.

Lindner, B., García-Ojalvo, J., Neiman, A., and S-G, L. (2004). Effects of noise in excitable systems. Phys. Rep. *392*, 321–424.

Liu, J., Martinez-Corral, R., Prindle, A., Lee, D.D., Larkin, J., Gabalda-Sagarra, M., Garcia-Ojalvo, J., and Süel, G.M. (2017). Coupling between distant biofilms and emergence of nutrient time-sharing. Science *356*, 638–642.

Mather, W.H., Hasty, J., Tsimring, L.S., and Williams, R.J. (2013). Translational cross talk in gene networks. Biophys. J. *104*, 2564–2572.

Mathur, M., Xiang, J.S., and Smolke, C.D. (2017). Mammalian synthetic biology for studying the cell. J. Cell Biol. *216*, 73–82.

McMillen, D., Kopell, N., Hasty, J., and Collins, J.J. (2002). Synchronizing genetic relaxation oscillators by intercell signaling. Proc. Natl. Acad. Sci. USA *99*, 679–684.

Niederholtmeyer, H., Sun, Z.Z., Hori, Y., Yeung, E., Verpoorte, A., Murray, R.M., and Maerkl, S.J. (2015). Rapid cell-free forward engineering of novel genetic ring oscillators. Elife *4*, e09771.

Nikolaev, E.V., and Sontag, E.D. (2016). Quorum-sensing synchronization of synthetic toggle switches: a design based on monotone dynamical systems theory. PLoS Comput. Biol. *12*, e1004881.

Otero-Muras, I., and Banga, J.R. (2016). Design principles of biological oscillators through optimization: forward and reverse analysis. PLoS One *11*, e0166867.

Panovska-Griffiths, J., Page, K.M., and Briscoe, J. (2013). A gene regulatory motif that generates oscillatory or multiway switch outputs. J. R. Soc. Interface *10*, 20120826.

Perez-Carrasco, R., Guerrero, P., Briscoe, J., and Page, K.M. (2016). Intrinsic noise profoundly alters the dynamics and steady state of morphogen-controlled bistable genetic switches. PLoS Comput. Biol. *12*, e1005154.

Pfeuty, B., and Kaneko, K. (2009). The combination of positive and negative feedback loops confers exquisite flexibility to biochemical switches. Phys. Biol. *6*, 046013.

Potvin-Trottier, L., Lord, N.D., Vinnicombe, G., and Paulsson, J. (2016). Synchronous long-term oscillations in a synthetic gene circuit. Nature *538*, 514–517.

Prindle, A., Selimkhanov, J., Li, H., Razinkov, I., Tsimring, L.S., and Hasty, J. (2014). Rapid and tunable post-translational coupling of genetic circuits. Nature *508*, 387–391.

Purcell, O., di Bernardo, M., Grierson, C.S., and Savery, N.J. (2011). A multi-functional synthetic gene network: a frequency multiplier, oscillator and switch. PLoS One *6*, e16140.

Purcell, O., Savery, N.J., Grierson, C.S., and di Bernardo, M. (2010). A comparative analysis of synthetic genetic oscillators. J. R. Soc. Interface *7*, 1503–1524.

Rubens, J.R., Selvaggio, G., and Lu, T.K. (2016). Synthetic mixed-signal computation in living cells. Nat. Commun. *7*, 11658.

Schaerli, Y., Munteanu, A., Gili, M., Cotterell, J., Sharpe, J., and Isalan, M. (2014). A unified design space of synthetic stripe-forming networks. Nat. Commun. *5*, 4905.

Scott, M., Gunderson, C.W., Mateescu, E.M., Zhang, Z., and Hwa, T. (2010). Interdependence of cell growth and gene expression: origins and consequences. Science *330*, 1099–1102.

Siuti, P., Yazbek, J., and Lu, T.K. (2013). Synthetic circuits integrating logic and memory in living cells. Nat. Biotechnol. *31*, 448–452.

Sohka, T., Heins, R.A., Phelan, R.M., Greisler, J.M., Townsend, C.A., and Ostermeier, M. (2009). An externally tunable bacterial band-pass filter. Proc. Natl. Acad. Sci. USA *106*, 10135–10140.

Sokolowski, T.R., Erdmann, T., and ten Wolde, P.R. (2012). Mutual repression enhances the steepness and precision of gene expression boundaries. PLoS Comput. Biol. *8*, e1002654.

Song, C., Phenix, H., Abedi, V., Scott, M., Ingalls, B.P., Kærn, M., and Perkins, T.J. (2010). Estimating the stochastic bifurcation structure of cellular networks. PLoS Comput. Biol. *6*, e1000699.

Strelkowa, N., and Barahona, M. (2010). Switchable genetic oscillator operating in quasi-stable mode. J. R. Soc. Interface *7*, 1071–1082.

Süel, G.M., Garcia-Ojalvo, J., Liberman, L.M., and Elowitz, M.B. (2006). An excitable gene regulatory circuit induces transient cellular differentiation. Nature *440*, 545–550.

Tan, C., Marguet, P., and You, L. (2009). Emergent bistability by a growth-modulating positive feedback circuit. Nat. Chem. Biol. *5*, 842–848.

Tian, T., and Burrage, K. (2006). Stochastic models for regulatory networks of the genetic toggle switch. Proc. Natl. Acad. Sci. USA *103*, 8372–8377.

Van Der Meer, J.R., and Belkin, S. (2010). Where microbiology meets microengineering: design and applications of reporter bacteria. Nat. Rev. Microbiol. *8*, 511–522.

Verd, B. (2016). EvoDevo in phase space: the dynamics of gap gene expression. PhD thesis (Pompeu Fabra University Barcelona).

Verd, B., Crombach, A., and Jaeger, J. (2014). Classification of transient behaviours in a time-dependent toggle switch model. BMC Syst. Biol. *8*, 43.

Webb, A.B., Lengyel, I.M., Jörg, D.J., Valentin, G., Jülicher, F., Morelli, L.G., and Oates, A.C. (2016). Persistence, period and precision of autonomous cellular oscillators from the zebrafish segmentation clock. Elife *5*, https://doi.org/10.7554/eLife.08438.

Woods, M.L., Leon, M., Perez-Carrasco, R., and Barnes, C.P. (2016). A statistical approach reveals designs for the most robust stochastic gene oscillators. ACS Synth. Biol. *5*, 459–470.

Xie, M., Ye, H., Wang, H., Charpin-El Hamri, G., Lormeau, C., Saxena, P., Stelling, J., and Fussenegger, M. (2016). β-Cell-mimetic designer cells provide closed-loop glycemic control. Science *354*, 1296–1301.

STAR★METHODS

KEY RESOURCES TABLE

REAGENT or RESOURCE	SOURCE	IDENTIFIER
Software and Algorithms		
ABC – SysBio	Liepe et al., 2010	http://www.theosysbio.bio.ic.ac.uk/resources/abc-sysbio/
PyDSTool	Clewley, 2012	http://www2.gsu.edu/~matrhc/PyDSTool.htm<br>RRID:SCR_014771

CONTACT FOR REAGENT AND RESOURCE SHARING

Further information and requests for resources and reagents should be directed to and will be fulfilled by the Lead Contact, Ruben Perez-Carrasco.

METHOD DETAILS

Non-dimensional Equations

The nondimensional equations [1] result from the dimensional Hill function regulation,

$$\begin{aligned}\frac{d\tilde{X}}{d\tilde{t}} &= \frac{\tilde{\alpha}_X + \tilde{\beta}_X S}{1 + S + \left(\tilde{Z}/\tilde{z}_X\right)^{n_{zx}}} - \tilde{\delta}_X \tilde{X}\\ \frac{d\tilde{Y}}{d\tilde{t}} &= \frac{\tilde{\alpha}_Y + \tilde{\beta}_Y S}{1 + S + \left(\tilde{X}/\tilde{x}_Y\right)^{n_{xy}}} - \tilde{\delta}_Y \tilde{Y}\\ \frac{d\tilde{Z}}{d\tilde{t}} &= \frac{\tilde{\alpha}_Z}{1 + \left(\tilde{X}/\tilde{x}_Z\right)^{n_{xz}} + \left(\tilde{Y}/\tilde{y}_Z\right)^{n_{yz}}} - \tilde{\delta}_Z Z.\end{aligned} \quad \text{(Equation 2)}$$

Measuring time in units of the degradation rate of protein X, all the temporal variables can be nondimensionalized as,

$$\delta_Y = \frac{\tilde{\delta}_Y}{\tilde{\delta}_X}, \quad \delta_Z = \frac{\tilde{\delta}_Z}{\tilde{\delta}_X}, \quad t = \tilde{t}\tilde{\delta}_X. \quad \text{(Equation 3)}$$

Similarly, concentrations and rates can be non-dimensionalized using the timescale of $\tilde{\delta}_X$ and the production rate $\tilde{\alpha}_Z$.

$$\alpha_X = \frac{\tilde{\alpha}_X}{\tilde{\alpha}_Z}, \ \alpha_Y = \frac{\tilde{\alpha}_Y}{\tilde{\alpha}_Z}, \ \beta_X = \frac{\tilde{\beta}_X}{\tilde{\alpha}_Z}, \ \beta_Y = \frac{\tilde{\beta}_Y}{\tilde{\alpha}_Z} \quad \text{(Equation 4)}$$

$$z_x = \frac{\tilde{z}_x \tilde{\delta}_X}{\tilde{\alpha}_Z}, \ x_y = \frac{\tilde{x}_y \tilde{\delta}_X}{\tilde{\alpha}_Z}, \ x_z = \frac{\tilde{x}_z \tilde{\delta}_X}{\tilde{\alpha}_Z}, \ y_z = \frac{\tilde{y}_z \tilde{\delta}_X}{\tilde{\alpha}_Z} \quad \text{(Equation 5)}$$

$$X = \frac{\tilde{X}\tilde{\delta}_X}{\tilde{\alpha}_Z}, \ Y = \frac{\tilde{Y}\tilde{\delta}_X}{\tilde{\alpha}_Z}, \ Z = \frac{\tilde{Z}\tilde{\delta}_X}{\tilde{\alpha}_Z} \quad \text{(Equation 6)}$$

The signal S is also measured in arbitrary units. Since S is a control parameter to control the dynamics properties of the system, the results will hold for any non-linear relationship between concentration of inducer and S.

For the stochastic Chemical Langevin Equation, the parameter Ω relates the non-dimensional expression levels with actual number of proteins (N_X, N_Y, N_Z) as,

$$N_X = \frac{X\tilde{\alpha}_Z\Omega}{\tilde{\delta}_X}, \quad N_Y = \frac{Y\tilde{\alpha}_Z\Omega}{\tilde{\delta}_X}, \quad N_Z = \frac{Z\tilde{\alpha}_Z\Omega}{\tilde{\delta}_X}. \quad \text{(Equation 7)}$$

Parameter Fitting

The parameter exploration was carried out using Bayesian sampling techniques through the Approximate Bayesian Computation (ABC) using ABC-SysBio software (Liepe et al., 2010). The score functions, $d()$, are minimal for the optimal behavior scored. They were designed to capture a change from stable steady state to oscillations. This was evaluated on trajectories for each parameter set under the induction of two different signal values S_{DC} and S_{AC}.

First the network is induced by a low signal $(S = S_{DC})$ for $\Delta t = 50$. Allowing a transient of $\Delta t = 30$ (see Figure S2), after which a constant response in time is scored:

$$d_{DC}(X(t)) = M_{DC} + 2\frac{\max(X(t)) - \min(X(t))}{\max(X(t)) + \min(X(t))} \quad \text{(Equation 8)}$$

Where M_{DC} is the number of minima found, penalising oscillations. The second term of d_{DC} penalises transients far from a steady expression.

The constant regime is perturbed by increasing the signal to a new value by multiplying it by a factor σ, $S_{AC} = S_{DC}\sigma$ $(\sigma > 1)$ applied for Δt = 100. The factor σ was also allowed to vary during the parameter exploration. During this second period, the goodness of the oscillations was evaluated favouring large oscillation amplitudes, and penalising a non-constant amplitude in time:

$$d_{AC}(X(t)) = \begin{cases} \frac{1}{M_{AC}} + 2 & M_{AC} < 4 \\ \left|\frac{A_M - A_{M-1}}{A_{M-1}}\right| + 2\frac{\min(X(t))}{\max(X(t)) + \min(X(t))} & M_{AC} > 4, \end{cases} \quad \text{(Equation 9)}$$

where M_{AC} is the number of maxima found after a transient of $\Delta t = 20$ region, and A_M and A_{M-1} are the amplitudes of the last and the previous to last full oscillations (see Figure S2). Both parameters S_{AC} and σ were treated as free parameters of the optimisation.

Finally, in order to reduce artefacts arising from the choice of high Hill exponents, all the Hill exponents were set to n = 2, varying only one exponent that is penalised to have higher values in circuits that already have a low score,

$$d_{Hill}(n_i) = \begin{cases} 2 & d_{AC} + d_{DC} > 2 \\ \left|\frac{2 - n_i}{3}\right| & d_{AC} + d_{DC} \le 2 \end{cases} \quad \text{(Equation 10)}$$

The distance used to infer the parameters used in the the current study (Table S1, and Figure S3) was,

$$d = d_{DC}(X) + d_{AC}(X) + d_{Hill}(n_{zx}), \quad \text{(Equation 11)}$$

where the minimisation was run for 20 generations of the ABC optimisation and the expected behavior started to arise beyond generation 10 of the ABC optimisation. To test possible overfitting resulting from the functions used, alternative functions were designed resulting in similar results analysed during different generations of the algorithm, some examples are shown in Table S2 where,

$$d_1 = d_{DC}^2(Y) + d_{AC}^2(Y) + d_{Hill}^2(n_{zx}), \quad \text{(Equation 12)}$$

$$d_2 = d_{DC}(X) + d_{AC}(Y) + d_{Hill}(n_{yz}). \quad \text{(Equation 13)}$$

Stochastic Expression

The stochastic dynamics of expression was studied using the Chemical Langevin equation resulting from taking into account the stochastic nature of the production and degradation events (Gillespie, 2000) as:

$$\dot{X} = f_X(Z, S) - X + \sqrt{f_X^2(Z, S) + X^2}\xi_X(t), \quad \text{(Equation 14)}$$

$$\dot{Y} = f_Y(X, S) - \delta_Y Y + \sqrt{f_Y^2(X, S) + \delta_Y^2 Y^2}\xi_Y(t),$$

$$\dot{Z} = f_Z(X, Y) - \delta_Z Z + \sqrt{f_Z^2(X, Y) + \delta_Z^2 Z^2}\xi_Z(t),$$

where f_X, f_Y, and f_Z are the production terms of equations.(1) and ξ_i are uncorrelated white Gaussian noises of zero mean and autocorrelation $\langle \xi_i(t)\xi_i(t')\rangle = \Omega^{-1}\delta(t-t')$, where $\delta(t-t')$ is Dirac's delta and Ω is the system volume, relating expression concentrations with number of proteins.

Cell Lattice Diffusion

Spatially extended simulations for the genetic expression propagation were carried out implementing an array of hexagonal cell of unit length. One or more of the proteins forming the AC-DC circuit are allowed to diffuse between neighbours using a discrete Laplacian that for gene X of the i-th cell reads

$$\dot{X}_i = D(\langle X \rangle_{\{i\}} - X_i), \quad \text{(Equation 15)}$$

where D is the intercellular diffusion coefficient and $\langle \cdot \rangle_{\{i\}}$ stands for the average expression of the target gene among of all the neighbouring cells of cell i.

Cell Systems, Volume *6*

Supplemental Information

Combining a Toggle Switch and a Repressilator within the AC-DC Circuit Generates Distinct Dynamical Behaviors

Ruben Perez-Carrasco, Chris P. Barnes, Yolanda Schaerli, Mark Isalan, James Briscoe, and Karen M. Page

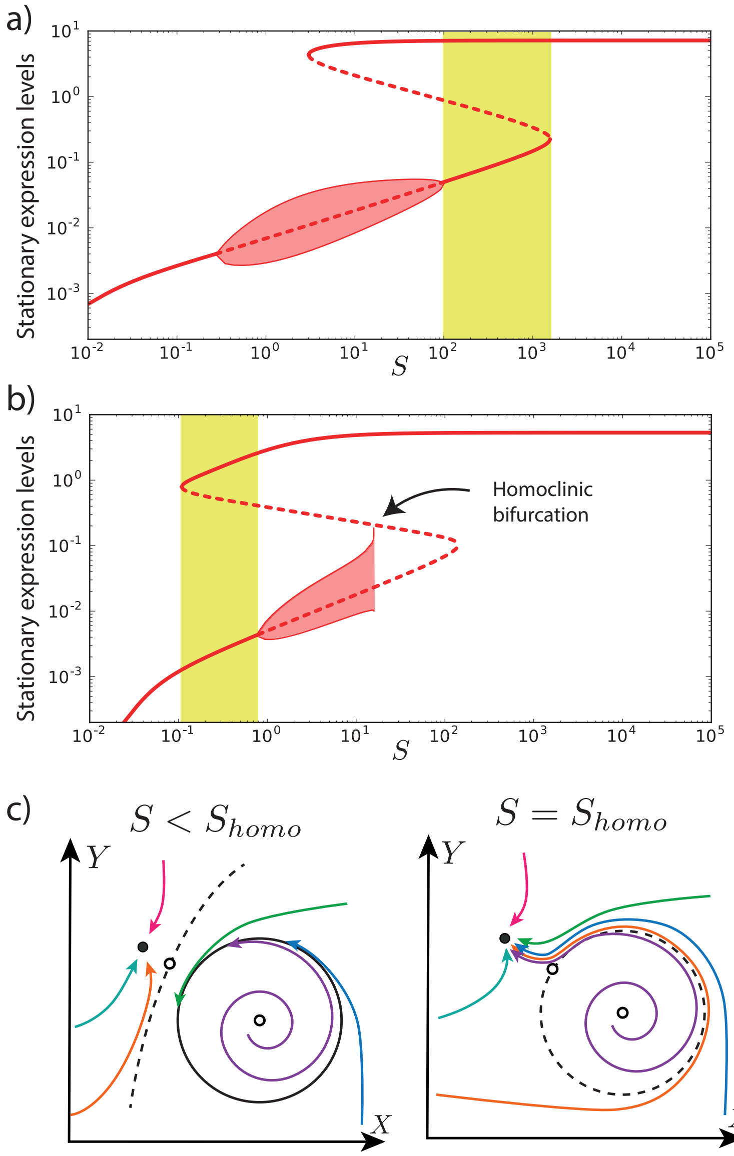

Figure S.1: Related to Figure 2. a) and b): Bifurcation diagrams showing bistability between two stable points for a wide range of signal (*yellow shaded zones*). This bistability is determined by the relative position of the saddle node bifurcations with respect to the Hopf bifurcation. This allows a bistable region between points, after (a) or before (b) the Hopf bifurcations. b) At high signals, stable oscillations can disappear through a homoclinic bifurcation instead of a Hopf bifurcation where the limit cycle collides with the unstable steady state at a signal $S_{homo} \simeq 16$. c) Scheme showing the attractor change at the homoclinic bifurcation. When the unstable saddle point (white point in the dashed line), collides with the limit cycle (solid circle), the cycle becomes unstable (dashed circle) and sustained oscillations are not available anymore. The only stable steady state remaining is the constant expression node that now attracts eventually all the expression trajectories except those starting precisely on the limit cycle. Parameters used for a) are $\alpha_x = 1.5$,$\alpha_y = 0.31$, $\beta_x = 7.17$, $\beta_y = 14.9$, $n_{xy} = 2.7$, $\delta_y = 1.31$, $\delta_z = 1.41$, $z_x = 1.75 \cdot 10^{-3}$, $x_y = 2.0 \cdot 10^{-3}$,$x_z = 0.19$, $y_z = 0.13$. Parameters used for b) are $\alpha_x = 0.486$,$\alpha_y = 0.252$, $\beta_x = 5.31$, $\beta_y = 6.2$, $n_{zx} = 2.8$, $\delta_y = 1.07$, $\delta_z = 2.0$, $z_x = 1.94 \cdot 10^{-3}$, $x_y = 1.4 \cdot 10^{-3}$,$x_z = 0.038$, $y_z = 0.097$.

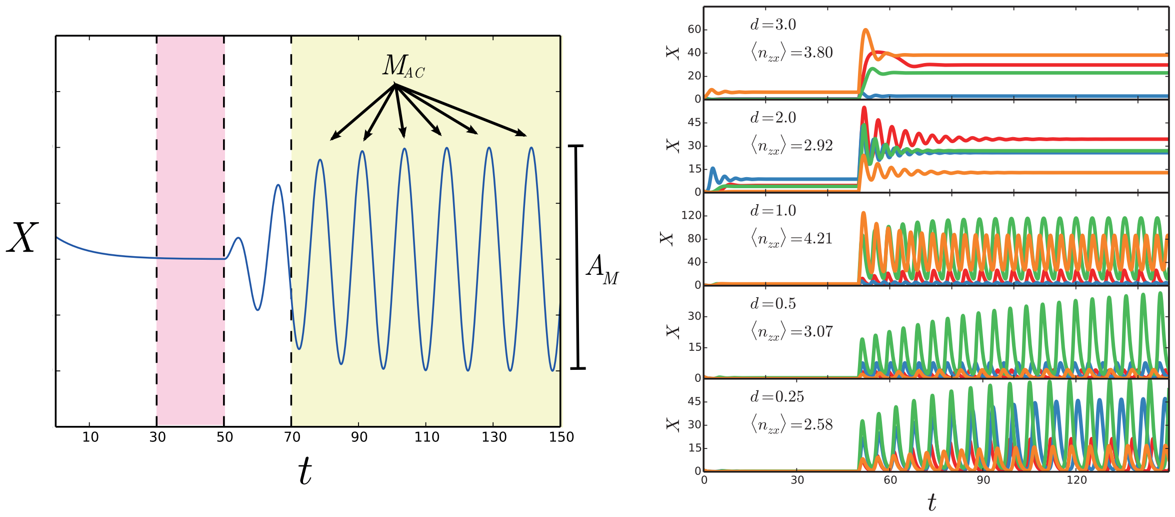

Figure S.2: Related to STAR Methods. Left) Target behavior used in the score function, evaluated at the shaded areas. The number of expression maxima at high signal M_{AC} and amplitude of the last oscillations A_M is used to compute the quality of oscillations. Right) Example results of behavior of the circuit under the two-signal protocol for different score results during the optimisation process.

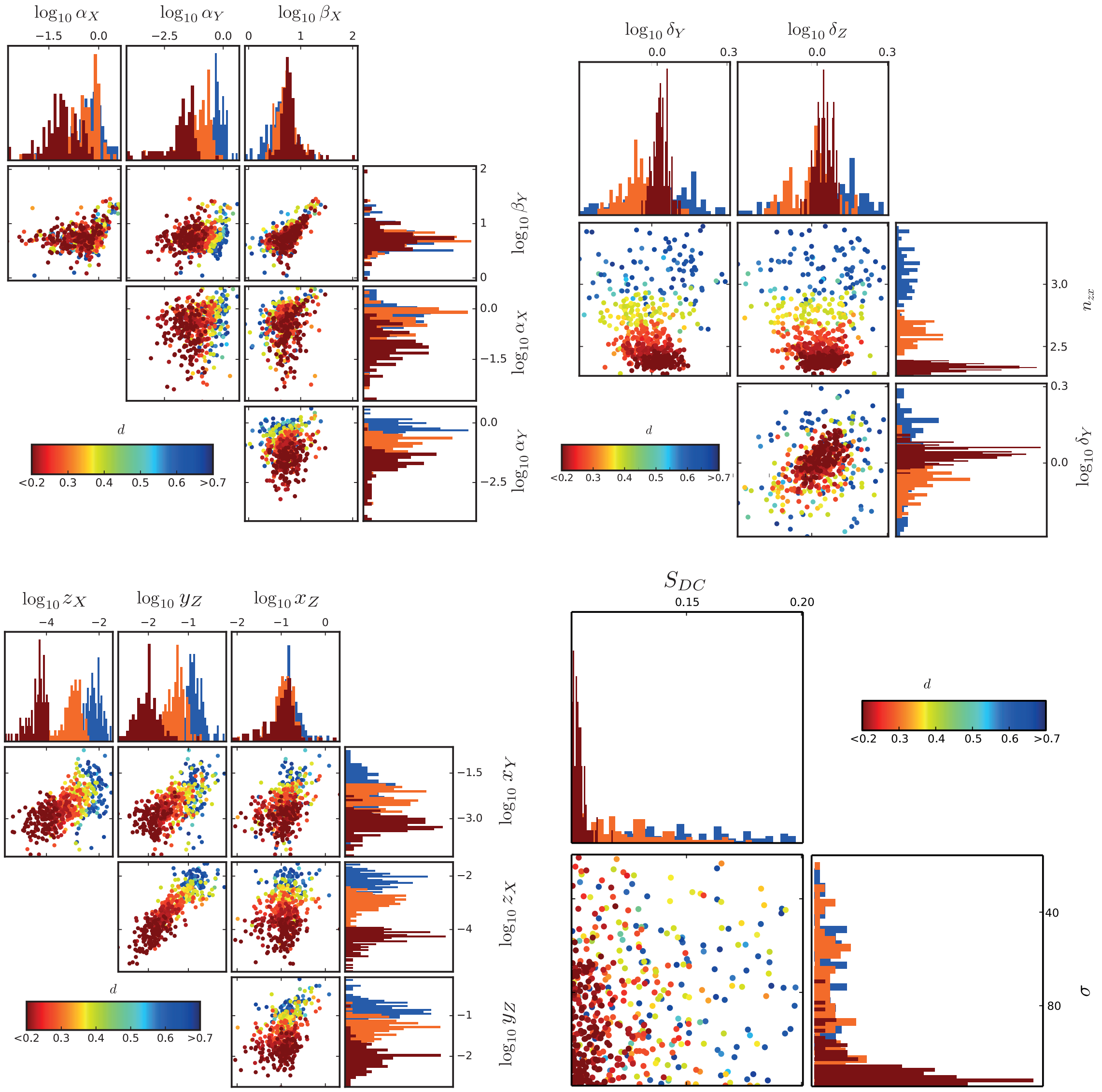

Figure S.3: Related to STAR Methods. Resulting parameter inference. Histograms correspond with the results of generations 10 (*blue*), 15 (*orange*) and 20 (*dark red*), colored by the average distance score of the generation. Scatter plots contain the sample used in generations 5,8,11,14, and 17. Parameters are grouped in 4 categories to make easier the comparison between different magnitudes.

Parameter	$\bar{x}\pm$ s.d	mode
α_X	$(15 \pm 14)10^{-2}$	$3.9 \cdot 10^{-2}$
α_Y	$(2.7 \pm 2.1)10^{-2}$	$4.3 \cdot 10^{-3}$
β_X	5.9 ± 0.8	6.1
β_Y	5.4 ± 1.2	5.7
δ_Y	1.07 ± 0.08	1.05
δ_Z	1.12 ± 0.09	1.04
z_X	$(6.4 \pm 4.3)10^{-5}$	$1.3 \cdot 10^{-5}$
y_Z	$(11 \pm 4)10^{-3}$	$11 \cdot 10^{-3}$
x_Z	$(12 \pm 5)10^{-2}$	$12 \cdot 10^{-2}$
x_Y	$(8.3 \pm 4.2)10^{-4}$	$7.9 \cdot 10^{-4}$
n_{zx}	2.34 ± 0.04	2.32

Table S.1: Related to Figure 2. Optimal parameters resulting from the last 300 points of the ABC fitting (generations 18-20). Each row shows the mean, standard deviation, and position of the peak of the distribution (mode). Rest of the Hill exponents were fixed to 2. For details see SI.

	d	$d^{(13)}$	d^*	d_1	d_2
α_X	0.57 ± 0.37	0.43 ± 0.31	0.64 ± 0.37	0.60 ± 0.39	0.60 ± 0.39
α_Y	0.21 ± 0.19	0.066 ± 0.055	0.18 ± 0.14	0.19 ± 0.14	0.23 ± 0.17
β_X	4.9 ± 1.3	5.4 ± 1.1	5.1 ± 1.5	4.9 ± 1.4	5.1 ± 1.3
β_Y	5.7 ± 1.0	5.2 ± 1.3	5.7 ± 1.2	5.6 ± 1.1	5.5 ± 1.1
δ_Y	0.93 ± 0.18	1.0 ± 0.1	0.94 ± 0.17	1.02 ± 0.27	1.00 ± 0.27
δ_Z	1.04 ± 0.22	1.1 ± 0.1	1.06 ± 0.21	1.05 ± 0.26	1.22 ± 0.27
z_X	$(1.7 \pm 1.4)10^{-3}$	$(2.7 \pm 2.2)10^{-4}$	$(1.6 \pm 1.5)10^{-3}$	$(3.0 \pm 2.1)10^{-3}$	$(6.0 \pm 5.8)10^{-4}$
y_Z	$(6.4 \pm 2.8)10{-}2$	$(2.0 \pm 0.9)10^{-2}$	$(6.1 \pm 2.5)10^{-2}$	$(9.0 \pm 3.8)10^{-2}$	$(4.6 \pm 2.4)10^{-2}$
x_Z	0.13 ± 0.05	0.11 ± 0.05	0.14 ± 0.05	0.14 ± 0.04	0.13 ± 0.05
x_Y	$(5.7 \pm 4.5)10^{-3}$	$(1.8 \pm 1.3)10^{-3}$	$(4.8 \pm 3.7)10^{-3}$	$(12.4 \pm 8)10^{-3}$	$(11 \pm 9)10^{-3}$
n	2.70 ± 0.15	2.44 ± 0.07	2.67 ± 0.14	2.42 ± 2.74	2.70 ± 0.16
σ	106 ± 44	135 ± 39	120 ± 42	114 ± 42	120 ± 40
S_{DC}	0.13 ± 0.13	0.22 ± 0.01	0.024 ± 25	0.132 ± 0.023	0.136 ± 0.023
d	0.33 ± 0.04	0.22 ± 0.01	0.32 ± 0.03	0.088 ± 0.023	0.359 ± 0.047

Table S.2: Related to STAR Methods. Inferred mean and standard deviation of the sampled parameter distribution for generations 8,9 and 10 (300 points). d^* is a second realisation of the inference using d to check the robustness of the optimisation. $d^{(13)}$ corresponds to generations 13, 14 and 15 for distance d. Alternative score functions d_1 and d_2 return similar parameter relationships. Parameter n is the varying Hill exponent corresponding to each score distance ($n = n_{zx}$ for d and d_1, and $n = n_{yz}$ for d_2), the rest of Hill exponents are fixed to 2).
